# Co-designing and evaluating a prenatal yoga intervention for ethnic minority women: a feasibility study

**DOI:** 10.1186/s40814-025-01667-9

**Published:** 2025-07-07

**Authors:** Carolina Estevao, Carola Chiarpenello, Winsze Kwok, Hemant Bhargav, Nishitha Jasti, Prabha Chandra, Shivarama Varambally, Carmine Pariante

**Affiliations:** 1 Institute of Psychiatry, Psychology and Neuroscience, Department of Psychological Medicine, King’s College London, London, UK; 2 Integrated Centre for Yoga, Department of Integrative Medicine, National Institute of Mental Health and Neurosciences, Bangalore, India; 3 Department of Psychiatry, National Institute of Mental Health and Neurosciences, Bangalore, India; 4 Biomedical Research Centre, National Institute for Health Research, South London and Maudsley NHS Foundation Trust and King’s College London, London, UK

**Keywords:** Pregnancy, Yoga, Ethnic minority, Prenatal depression, Prenatal anxiety

## Abstract

**Background:**

Prenatal depression is the greatest risk factor for postnatal depression and subsequently, impaired mother-infant attachment. Prenatal yoga may improve both mental and physical health during pregnancy and support mother–fetal attachment. However, its integration into perinatal care remains limited due to a lack of standardization. This study primarily assessed the feasibility of a prenatal yoga module co-developed by King’s College London and the National Institute of Mental Health and Neurosciences (NIMHANS). This study aimed to evaluate the feasibility of the first rigorously designed, scientifically backed prenatal yoga module for mental health and mother–fetal attachment.

**Methods:**

We codeveloped the PRENAYOGA intervention through Preliminary Patient and Public Involvement (PPI) and a 3-month collaboration project with NIMHANS. The final yoga module consisted of biweekly 1-h sessions for 8 weeks in South London, with 15 ethnic minority women. Data were collected at baseline, 4 weeks, and 8 weeks. Feasibility outcomes, including intervention acceptability, appropriateness, and attendance, were assessed using validated measures and focus group data. Clinical exploratory outcomes, such as mental health and maternal–fetal attachment, quality of life, social support, and self-efficacy were also evaluated.

**Results:**

The findings demonstrated the acceptability and appropriateness of the adapted yoga module among participants and stakeholders. Attendance and attrition rates alongside qualitative analyses highlighted both barriers and enablers to sustained engagement. Participants reported enhanced physical and mental health, flexibility, and relief from physical ailments. Insights into session frequency and duration suggested that two 1-h sessions per week for 8 weeks are a viable model, with 80% attending at least once weekly. Yoga teachers highlighted the community-building aspect and adapted sessions to participants’ unique needs. Preliminary clinical findings suggested improvements in mental health and maternal–fetal attachment, though these require further investigation.

**Conclusions:**

These preliminary results indicate the potential benefit of rigorously developed prenatal yoga for ethnic minority populations. These findings support the need for larger trials to evaluate the module’s clinical effectiveness and scalability; such research would contribute to evidence-based prenatal care for underrepresented populations.

**Trial registration:**

ClinicalTrial.gov Identifier: NCT05824208.

**Supplementary Information:**

The online version contains supplementary material available at 10.1186/s40814-025-01667-9

## Key messages

• Initial uncertainties included the adaptability of prenatal yoga for the target population in London, engagement and attendance consistency, and the intervention’s potential impact on well-being and mother–fetal attachment.

• High acceptability and positive health outcomes were reported, with a sustainable session model indicating regular attendance and community building. Exploratory clinical results showed potential improvements in depressive symptoms, stress, and overall quality of life.

• The findings further build on the need for a randomized clinical trial to explore the effects of prenatal yoga on depression and mother–fetal attachment.

## Background

Depression and anxiety are the most prevalent psychiatric disorders in the perinatal period [1]. A recent report found that nearly 50% of new mothers in London (where 40% of the population is of ethnic minority background) displayed symptoms of postnatal depression (PND) [2].

Additionally, a 2022 report by the London School of Economics (LSE) found that treating maternal mental illness could save the National Health Service (NHS) £52 million over 10 years. In 2014, the LSE calculated that two-thirds of that cost is linked to adverse child development [3].

Prenatal depression results in adverse outcomes for the mother and infant [4, 5]. Indeed, depression in the postnatal period negatively impacts the emotional relationship and attachment between the mother and the child. The major risk for developing postnatal depression is a history of depression, either in the lifetime or during pregnancy [6–8].

Several studies highlight that prenatal depression rates are higher in ethnic minority women [9, 10], who are also likely to experience a lower quality of healthcare than White women [11]. This reduced access often results in poorer health outcomes and reports of worse experiences with NHS services; UK maternal mortality data confirm this [12]. A recent survey in the UK showed that ethnic minority women are more likely to access services late in pregnancy, experience obstetric complications, and are less likely to attend prenatal classes than White women [13]. Another UK-based study revealed that Asian and Black women are less likely to be offered prenatal classes [14]. Unsurprisingly, the inclusion of ethnically diverse women in perinatal depression studies is negligible, mirroring the disproportionately low ethnic minority women’s (particularly Black Caribbean) representation in perinatal mental health therapeutic settings [15].

Preliminary evidence indicates that prenatal yoga reduces anxiety and depression [16, 17] and improves mother–fetal attachment [18–22]. Systematic reviews and meta-analyses confirm significant reductions in anxiety and depressive symptoms among pregnant individuals practicing yoga, with small to moderate effect sizes [23, 24]. Additionally, prenatal yoga has been shown to enhance maternal–fetal bonding by fostering mindfulness and emotional regulation, particularly through targeted breathing exercises and meditation practices [18–20].

Ethnic minority groups have been identified as particularly receptive to mind–body interventions such as yoga, which align with cultural preferences for holistic, community-based health practices [25, 26]. However, the focus of most of these studies is context-based, frequently focusing on African-American populations, which may not be representative of other minority groups outside of the USA. However, these interventions often provide an accessible and culturally sensitivattended a focus group at week e alternative to traditional prenatal classes, addressing barriers such as language, cost, and perceived discrimination in healthcare settings [27]. Furthermore, participation in mind–body programs tailored to specific cultural or ethnic groups has been linked to higher engagement and sustained attendance, reinforcing the importance of community-oriented approaches in promoting perinatal mental health [28, 29].

While prenatal yoga has shown promise in promoting maternal well-being, its adaptation for ethnic minority populations remains underexplored. This study aimed to fill this gap by developing and evaluating a scientifically sound co-developed prenatal yoga intervention tailored to the needs and preferences of pregnant women from ethnic minority backgrounds in London. By focusing on feasibility, acceptability, and potential mental health benefits, this study seeks to address disparities in access to holistic prenatal care and provide insights into effective strategies for improving maternal well-being in underserved communities.

The PRENAYOGA study, described in this paper, included initial engagement with patients and the public through a focus group and a survey. The second phase was conducted in partnership with the Department of Integrative Medicine at the National Institute of Mental Health and Neurosciences (NIMHANS) and stakeholders in the prenatal health space. The third phase was a feasibility study with 15 ethnic minority participants in a yoga studio in London, followed by module adaptations resulting from the feasibility study findings.

### Study aim and objectives

The broad aim of this study is to evaluate the acceptability and feasibility of a co-designed prenatal yoga intervention (PRENAYOGA), focusing on its potential to improve mental health, and maternal–fetal attachment, while unveiling barriers to engagement within this underrepresented population.

Primary feasibility objective: To assess the acceptability of the intervention.

Secondary feasibility objectives:

- To assess the appropriateness and feasibility of the intervention.
- To evaluate attendance and attrition rates.
- To explore the lived experiences of pregnant women from ethnic minority backgrounds participating in the study, including those who discontinued or attended fewer than 50% of sessions.
- To examine stakeholder perspectives on fidelity, sustainability, and scalability.

Secondary exploratory clinical objectives: To explore potential improvements in mental health, quality of life, social support, self-efficacy, and maternal–fetal attachment.

## Methods

### Preliminary patient and public involvement (PPI) work

In preparation for the module development, we conducted a preliminary investigation in London with an independent group of ethnic minority *multigravida* women:

i. We consulted with 3 ethnic minority women who had experienced prenatal depression to gauge their interest in participating in weekly prenatal yoga sessions. They preferred small classes, held in the evenings or on weekends, lasting no more than 1 hour, and within a 30-minute commute.
ii. We interviewed 4 ethnic minority women who practised prenatal yoga and had mixed views on face-to-face versus online yoga sessions, seeing advantages in both approaches. Some suggested aligning research activities with doctor appointments or outside working hours. One participant highlighted the importance of free classes and shared the therapeutic benefits of prenatal yoga she personally experienced. These four participants agreed on a small class size and a duration of 1–2 hours.
iii. A survey, including participants from groups i and ii and 2 additional women (*n*=9), showed that 89% were willing to participate in online assessments and 100% complete self-reporting questionnaires.

The preliminary PPI work, although informative, involved a small and potentially self-selecting group of women who may have been predisposed to engaging with yoga-based interventions. As such, the perspectives gathered may not fully represent the broader diversity of views within the target population. Considering the caveats, these findings informed the development of the PRENAYOGA protocol and schedule in collaboration with NIMHANS.

### Results of literature review to inform module development

To create a framework of potential components for an evidence-based yoga module for pregnancy for mental health and maternal–fetal attachment, a review of the literature was performed. Research question: What are the components of effective prenatal yoga interventions for attachment and mental health difficulties in pregnancy?—as per clinical studies published up to December 2022. An Internet-based search was performed through PubMed, Google Scholar, and Scopus from all available articles until December 05, 2022 (Table 1).

**Table 1:** Database and search terms for literature review

Database	Search terms	Number of results
PubMed	((yoga[Title/Abstract]) AND (pregnant[Title/Abstract])) OR ((yoga[Title/Abstract]) AND (pregnancy[Title/Abstract])) OR ((yoga[Title/Abstract]) AND (antenatal[Title/Abstract])) OR ((yoga[Title/Abstract]) AND (prenatal[Title/Abstract])). Results filtered by: Clinical Trial, Randomized Controlled Trial and Language: English	53
Scopus	the following terms were used, before filtering by Source Type: Journal, Document type: Article and Review, and Language: English: TITLE-ABS-KEY ("yoga") AND TITLE-ABS-KEY ("pregnancy"OR"pregnant"OR"prenatal"OR"antenatal")	381
Google Scholar	yoga pregnancy, OR pregnant, OR prenatal, OR antenatal. Results filtered by: not citation only	37

The search results were exported to Zotero and refined based on inclusion criteria: English language, clinical trials/studies, peer-reviewed publications (excluding theses, book chapters, and conference abstracts), quantitative outcomes reporting clinical effectiveness, a specified prenatal yoga protocol (including at least four asanas), and at least one self-report measure of stress, anxiety, depression, or maternal–fetal attachment. Both physical exercise-based yoga (asanas) and integrated yoga (including pranayama and meditation) were included. Participants included both depressed individuals and healthy controls.

The review focused primarily on asanas frequently used in prenatal yoga studies for well-being and maternal–fetal attachment, while secondarily examining study design, sample size, eligibility criteria, intervention details, and outcomes.

**Fig. 1:**
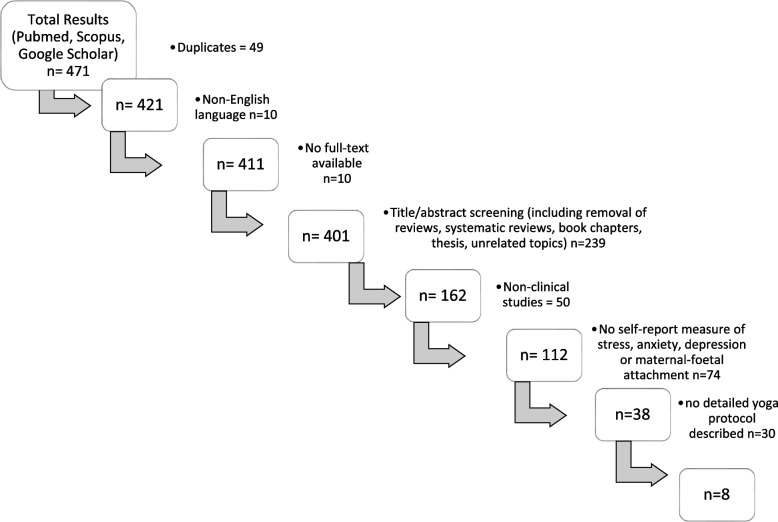
Flow chart of study selection for literature review to inform the module development

Of 471 initial results from PubMed, Scopus, and Google Scholar, only eight studies met the criteria. Exclusions included duplicates (*n* = 49), non-English texts (*n* = 10), unavailable full texts (*n* = 10), unrelated abstracts/titles (*n* = 239), non-peer–reviewed publications (e.g., theses), reviews, and non-clinical studies (*n* = 50). Of the 112 remaining studies, 74 lacked relevant outcomes or efficacy data, and 30 failed to describe yoga interventions sufficiently (e.g., fewer than four asanas or no class sequence). The final eight studies (815 participants) met all criteria for analysis (Fig. 1).

#### Sample size and study designs

The sample sizes in the eight studies analyzed ranged from 63 to 181 participants, with an average of 102 participants per study and a total of 815 participants in total. The sample size variability is evidenced by the study designs, with six randomized controlled trials (RCT), one convenience sampling study, and one controlled study.

#### Eligibility criteria and gestational age

The eligibility was heterogeneous, with studies defining their sample of interest by “low-risk pregnancies” (*n* = 5, low-risk pregnancies) [18, 19, 33]. The definition of pregnancy risk was not always described. Interestingly, one study designed a prenatal yoga intervention for high-risk pregnancies requiring bedrest [34]. While one study excluded a history of mental health complications [19], three studies recruited a clinical population with prenatal depression [23, 33, 35].

Studies mostly recruited women with singleton pregnancies (twin pregnancies were considered ineligible apart from three studies) [19, 33, 35].

The gestational age of women recruited ranged from 14 to 42 weeks, with an average of 28 weeks, with all studies recruiting women at least in the second trimester of pregnancy. Only one study recruited women in the third trimester [34] due to the nature of the sample (high-risk pregnancies in bedrest).

#### Control group

All interventions had a comparator group. While most used the standard of care as a comparator to the yoga group, one study used a social support group [35], another used massage therapy alongside standard prenatal care [33], and two used standard prenatal exercises [36, 37].

#### Yoga schedule

The description of the yoga schedules varied greatly; while some programs were time-limited to 8 weeks [18] and 12 weeks [23, 33, 35], others expected yoga participants to attend at least 8 h of practice (six sessions) [19], and the remaining studies suggested practice until birth.

The duration of the classes also varied from 20-min weekly sessions [33, 35], 30-min weekly sessions [34], 40–45 min weekly sessions [18], 75-min weekly classes [23], 90-min sessions [19], to 120-min sessions three times per week, followed by home practices of 1 h [36, 37].

Classes were practiced in small groups, from 4 to 10 women, with one study reporting individual sessions [34].

#### Class sequence

All the classes described in the studies reviewed involved *asana* practice. In addition, most studies described breathing exercises/*pranayama* [18, 19, 23, 34, 37] (either in the form of synchronized breath and joint exercises and breathing or specific pranayamas), meditation [18, 37], including guided visualization [34, 36], and final relaxation [19, 23, 34, 36]. Some classes specified warming-up exercises [19], centering [19, 23], counseling [36] or lectures [37], affirmations [19], and mother–baby communication [18].

#### Asana practice

While some studies referred to specific *asanas* in their Sanskrit names, others did so in English. The *asanas* described in the eight studies above can be organized into the following categories:

Warm-up/joint loosening: *kantasanchalana* (neck rolls close up) [34]; prone *urdhva hastasana* (upward salute) [34]; *sukhasana* (easy seat) rotations and side stretches [23, 33–35]; *parivrtta sukhasana* (revolved easy pose) [23, 33, 35]; neck/shoulder rolls [23]; thoracic/lumbar stretches [34]; pelvic rocking [23]; foot pumps [34]; *bitilasana/marjariasana* (cat-cow pose) [18, 23, 33, 35]; *bitilasana* (cow pose) [18, 33]; *dandayamana bharmanasana* (bird-dog) [18, 33, 35]; sun breaths [23]; hip circles [23]; *hasta ayama svasanam* (hands-in and hands-out breathing) [36]; *hasta vistara svasan* (hands stretch breathing) [36]; *gulpha vistara svasanam* (ankle stretch breathing) [36]; *vyāghra śvasanam* (tiger breathing) [36]; and *setu bandha śvasanam* (bridge pose) [36].

Sun Salutations: pregnancy *surya namaskar* (sun salutation) [33], including *ashtanga namaskara* (eight-limbed pose) to *balasana* (child’s pose) [23].

Standing and balancing: *tadasana* I (mountain pose I) [18, 23, 36, 37], *tadasana* II (mountain pose II) [18], *trikonasana* (triangle pose) [33, 35–37], *uttihita trikonasana* (extended triangle pose) [18], *parivrtta trikonasana* (revolved triangle pose) [33], *natarajasana* (dancer’s pose) [18], standing *garudasana* (eagle) [18], *uttanasana* (standing forward bend) [18], *virabhadrasana* I (warrior I) [18, 23, 33, 35], *virabhadrasana* II (warrior II) [23, 33, 35], *viparita virabhadrasana* (reverse warrior pose) [35], kneeling *virabhadrasana* (warrior) [33, 35], *anjaneyasana* (low lunge) [35], *saaras pakshiasana* (stork bird pose) [33, 35], *vrksasana* (tree pose) [23, 33, 35], *utthita parshvakonasana* (pyramid pose) [35], and *ardhakati chakrasana* (half waist wheel pose) [36, 37].

Sitting and on knees: *sukhasana* (easy seat) [33, 35], *padmasana* (lotus pose) [18], *parivritta sukhasana* (revolved easy pose) [18], *garudasana* (eagle pose) [18, 33, 35], *paschimoottanasana* (seated forward fold) [18, 33], *baddha konasana* (bound angle pose) [18, 33, 35–37], *upavistha konasana* (wide-angle seated forward bend) [18, 36, 37], *parsva upavistha konasana* (seated side stretch) [35], *vajrasana* (diamond pose) [18, 36, 37], *janu sirsasana* (head to knee pose) [18], *gomukasana* (cow face pose) [33, 35], *utthita ashwa sanchalanasana* (high lunge) [33], *malasana* (yogic squat) [23, 36, 37], *ardha uttanasana* (half forward fold) [23], *siddhasana* (accomplished pose) [36, 37], *vakrasana* (spinal twist) [36, 37], and *pranamasana* (prayer pose) [35].

Prone, supine, and restorative poses, *balasana* (child’s pose) [33]; *supta matsyendrasana* (supine spinal twist) [35]; *supta baddha konasana* (reclined bound angle pose) [23]; *viparita karani* (legs up the wall pose) [23, 36, 37]; and *ardha pavanamuktasana* (half wind relieving pose) [36, 37].

Note: for consistency and reverence for the origins of the practice, when *asana* names were described in English, the Sanskrit translation was used when possible.

#### Outcomes

Given the focus on mental health, wellbeing, and attachment outcomes of the prenatal yoga practices described in the studies, we have highlighted the main outcomes of interest below:

- Short-term effects on depression and anxiety (within-session reductions) using the Profile of Mood States (POMS) scale and anxiety using the State Anxiety Inventory (STAI) on the first and last session (in comparison with the control group) [35].
- Long-term (baseline to last session) decreases in the following:
  o Depression using the Edinburgh Postnatal Depression Scale (EPDS) [23, 33, 35], Center for Epidemiological Studies Depression Scale (CES-D) [33, 35], the POMS [33, 35], Hospital Anxiety and Depression Scale (HADS) [34, 37], and the Patient Health Questionnaire-9 (PHQ-9) [23];
  o Anxiety using the STAI [23, 33, 35, 37] and Hospital Anxiety and Depression Scale (HADS) [34];
  o Stress using the Perceived Stress Scale (PSS) [23, 36].
- Two studies found increased fetal attachment using the prenatal attachment inventory (PAI) [18, 19] and the Maternal–Fetal Attachment Scale (MFAS) [23].

#### Limitations

One of the studies offered a class sequence (prenatal sun salutations, standing, kneeling, sitting poses, supine/restorative poses), but no specific *asanas* within each category were described [19] and another study described modified *asanas* for high-risk, bed-rest patients but no specific *asana* names were given [34].

One of the studies [18] highlighted that their sample was of highly educated women, a common finding in clinical studies that hinders the generalization of findings. In line with this limitation, another study found that education level and engagement with health services (number of fetal ultrasounds), two characteristics that indicate a higher socio-economic sample, impacted attachment [19].

In conclusion, the above literature review has revealed that despite the research resulting in 471 potential publications on the topic of pregnancy yoga, only eight studies provided details of pregnancy yoga poses that could inform the development of an evidence-based yoga module.

Given that the research question was based around the physical yoga poses (*asanas)*, the primary aim of this literature review focused on which *asanas* have been repeatedly reported in yoga studies that focused on mental health and/or attachment outcomes. The *asanas* that were more frequently described (more than one study in this review) were as follows:

Loosening exercises: *sukhasana* rotations and side stretches [23, 33–35], *parivrtta sukhasana* [23, 33, 35], *bitilasana/marjariasana* [18, 23, 33, 35], *bitilasana* [18, 33], and *dandayamana bharmanasana* [18, 33, 35].

Standing and balancing poses: *tadasana* I [18, 23, 36, 37], *trikonasana* [33, 35–37], *virabhadrasana* I [18, 23, 33, 35], *virabhadrasana* II [23, 33, 35], kneeling *virabhadrasana* [33, 35], *saaras pakshiasana* [33, 35], *vrksasana* [23, 33, 35], and *ardhakati chakrasana* [36, 37].

Sitting and on knees: *sukhasana* [33, 35], *garudasana* [18, 33, 35], *paschimoottanasana* [18, 33], *baddha konasana* [18, 33, 35–37], *upavistha konasana* [18, 36, 37], *vajrasana* [18, 36, 37], *gomukasana* [33, 35], *malasana* [23, 36, 37], *siddhasana* [36, 37], and *vakrasana* [36, 37].

Prone, supine, and restorative poses: *viparita karani* [23, 36, 37] and *ardha pavanamuktasana* [36, 37].

As a secondary aim, other aspects were explored, and the following conclusions were reached:

- Studies selected in this review, recruited women with an average of 28 weeks of gestation, with all studies recruiting from at least 14 weeks of gestation.
- The minimum practice was 8 weeks, while some studies delivered a prenatal yoga intervention up until birth.
- The duration of classes ranged from 20 to 120 min weekly, with two studies suggesting home practice.
- Class size was an average of seven women.
- Most studies described breathing exercises (in the “warm-up” portion of the class or as formal *pranayamas*).
- Some included meditation, guided visualizations, final relaxation, and centering.

Despite the growing body of research on the benefits of yoga during pregnancy, there is currently a lack of evidence on specific yoga module details, such as the optimal frequency, duration, gestational age, and specific details of the physical practice (*asana, pranayama*, meditation practices). Additionally, many existing studies have small sample sizes and are not adequately controlled, making it difficult to draw definitive conclusions about the effectiveness of yoga for pregnancy.

Following this review, in response to the absence of standardized prenatal yoga modules, inconsistency in reported outcome measures, and a lack of clearly defined protocols, we developed a structured yoga module specifically tailored for prenatal use.

### Prenatal yoga module development

Following a literature review on the yoga poses (*asanas*) reported in studies for prenatal mental health and/or maternal–fetal attachment, we interviewed 12 prenatal experts (yoga therapists, academics, perinatal psychiatrists, psychologists, and a doctor of obstetrics and gynecology), in collaboration with the National Institute of Mental Health and Neurosciences (NIMHANS) in Bangalore, India (institution of authors HB, NJ, PC, and SV), on the elements they believed a prenatal yoga module should encompass.

We compiled the results of the literature review and elements mentioned by the experts into a module with loosening (warm-up) exercises, poses (*asanas*), breathwork (*pranayama*), chanting, meditation, and yogic philosophy/counseling. This module was rated by 11 further independent experts (academics, yoga researchers, yoga therapists, and a medical director) (see Table 8 in Appendix I).

The agreement was assessed by calculating the content validity ratio using Lawshe’s formula [38]. Based on the number of experts consulted, the final yoga module retained practices with a CVR score above 0.81. A final module consisting of 38 elements was devised and a booklet for the training of the yoga teachers was created and tested in the PRENAYOGA study.

### Study design

PRENAYOGA is a single-arm feasibility study of a prenatal yoga intervention. The intervention was delivered biweekly in a yoga studio in South London for 8 weeks, from April to June 2023. The studio provided participants with yoga mats, blocks, bolsters, and straps. The classes were adapted to pregnancy and for all ability levels; lessons included loosening exercises (warm-up exercises), simple postures/*asanas* (standing, balancing, sitting, kneeling, supine, and restorative poses), and breathwork/*pranayama* exercises, meditation, and yogic counseling, as per the yoga module designed (see Appendix VIII). Internationally certified and insured yoga teachers, trained on the yoga module (two 45-min training sessions, including a 45-min refresher and continuous access to the module booklet) delivered the classes, accompanied by soft instrumental music of their choice.

### Study outcomes

The following outcome measures, linked to each study objective were collected (Table 2).

**Table 2:** Objectives and Outcome Measures

Objective	Outcome measure	Type of measure	Collected from
**Primary feasibility objective**			
Acceptability of the intervention	Acceptability of Intervention Measure (AIM), focus group, and interviews	Quantitative (AIM), qualitative (focus group/interviews)	Participants, stakeholders
**Secondary feasibility objectives**			
Appropriateness of the intervention	Intervention Appropriateness Measure (IAM), focus group, and interviews	Quantitative (IAM), Qualitative (focus group/interviews)	Participants, stakeholders
Feasibility of the intervention	Feasibility of Intervention Measure (FIM), focus group, and interviews	Quantitative (FIM), Qualitative (focus group/interviews)	Participants, stakeholders
Attendance rates	Weekly session attendance monitoring	Quantitative	Participants
Attrition rates	Participant dropout tracking	Quantitative	Participants
Lived experience of pregnant women from ethnic minority backgrounds in the study	Focus group and interviews (script in Appendix II)	Qualitative	Participants
Lived experience of pregnant women from ethnic minority backgrounds in the study (who discontinued the intervention of attended < 50% of the sessions)	Interviews (script in Appendix II)	Qualitative	Participants who discontinued the intervention of attended < 50% of the sessions
Fidelity, sustainability, and scalability factors	Interviews (script in Appendix II)	Qualitative	Stakeholders
**Secondary exploratory clinical objectives**			
Mental health improvements	Edinburgh Postnatal Depression Scale (EPDS), Beck Depression Inventory (BDI), State-Trait Anxiety Inventory (STAI), and Perceived Stress Scale (PSS)	Quantitative	Participants
Quality of life	EQ5D-5L (quality of life measure)	Quantitative	Participants
Social support	Multidimensional Scale of Perceived Social Support (MSPSS)	Quantitative	Participants
Self-efficacy	Short General Self-Efficacy Scale (GSE-6)	Quantitative	Participants
Mother–fetal attachment*	Prenatal Attachment Inventory (PAI), Maternal Foetal Attachment Scale (MFAS), and Maternal Antenatal Attachment Scale (MAAS)	Quantitative	Participants

^*^Three validated attachment measures (PAI, MFAS, MAAS) were used to capture different dimensions of maternal–foetal attachment, recognising that each scale has distinct psychometric properties and focus areas (e.g., emotional bonding vs behavioural expression). Their inclusion allowed for triangulation of results and enhanced construct validity

Feasibility benchmarks:

- **Acceptability, appropriateness, and feasibility**: We have set a target total average score of 4 out of 5 on the AIM, IAM, and FIM, indicating a high perception of acceptability, appropriateness, and feasibility among participants.
- **Attendance**: ≥ 80% weekly attendance, reflecting attendance rates achieved in a comparable prenatal yoga intervention [30].
- **Attrition**: ≤ 20% dropout rate (max of three participants), aligning with retention rates reported in similar studies [31].
- **Quality of life**: ≥ 10% improvement on EQ-5D-5L (EQ-VAS), recognizing, however, that previous research suggests that quality of life declines with gestational age [32].

### Recruitment methods and data collection

Participants provided informed consent and completed feasibility, mental health, attachment, self-efficacy, social support, and quality of life questionnaires via email at baseline, week 4, and week 8 (end of intervention).

Two focus groups, led by one of the researchers (WK), took place during the last week of the sessions with attendees. The topics explored included expectations of yoga, experiences of the intervention, facilitators and barriers, and views on future research (see topic guides in Appendix II).

Separately, we interviewed those who withdrew (*n* = 1) or attended < 50% of sessions to gain insights into factors that may lead to disengagement (see Appendix II). Finally, we interviewed the yoga teachers (stakeholders) to gather insights into the practical implementation and adaptation of the prenatal yoga module (fidelity of the delivery) (see Appendix II).

The schedule of data collection can be seen in Tables 3 and 4 below:

**Table 3:** Feasibility measures schedule

	Baseline	Week 4	End of study	
	Q	Q	Q	FG/I
Acceptability of Intervention Measure (AIM)			x	x
Intervention Appropriateness Measure (IAM)			x	x
Feasibility of Intervention Measure (FIM)			x	x
Attendance rates	x (weekly)			
Drop-out rates	x (continuous)			

^*Q*, questionnaire^

^*FG*, focus group^

^*I*, interview (semi−structured)^

**Table 4:** Clinical measure schedule

	Baseline			Week 4	End of study	
	Visit	Q	1^st^ S	Q	Q	8^th^ S
Demographics & quality of life						
Baseline demographics	x					
EQ5D-5L		x		x	x	
Mental health						
Edinburgh postnatal depression scale (EPDS)		x		x	X	
Beck depression inventory (BDI)		x		x	X	
State-trait anxiety inventory (STAI)		x		x	x	
Perceived stress scale (PSS)		x		x	x	
Social						
Short general self-efficacy scale (GSE-6)		x		x	x	
Multidimensional scale of perceived social support (MSPSS)		x		x	x	
Attachment						
Prenatal Attachment Inventory (PAI)		x		x	x	
Maternal Foetal Attachment Scale (MFAS)		x		x	x	
Maternal Antenatal Attachment Scale (MAAS)		x		x	x	
Qualitative						
Focus group						x
Interviews (withdrawals, < 50% of sessions and stakeholders)						x

^*S*, session^

^*Q*, questionnaire^

^*PP*, postpartum^

### Sample size justification

For the PRENAYOGA study, a sample size of 15 participants was selected. This aligns with recommendations in feasibility study methodology, which suggest that sample sizes of at least 12 participants are sufficient for preliminary feasibility outcomes, as well as for identifying potential issues in study procedures without exposing an excessive number of participants to an intervention that is still under evaluation [39]. Given our mixed-methods design, this sample size allowed us to gather both quantitative indicators and qualitative insights, maintaining a low participant burden. Assuming an expected retention rate of 80%, the 95% confidence interval around this estimate for a sample size of *N* = 15 is approximately 55–95%. This interval reflects the uncertainty associated with small samples and the emphasis on feasibility rather than definitive conclusions about PRENAYOGA’s effectiveness.

### Recruitment

Recruitment efforts were run at the community level, including advertisements in local libraries, shops, cafes, faith spaces (churches, mosques, temples), pharmacies, community centers, and local shops. Additionally, we advertised on Instagram, Facebook, and parenthood apps (such as Happity).

Participants expressed their interest by completing a short webform and were then contacted via phone or e-mail by one of the researchers (CE, CC, or WK). During the initial call, which lasted between 10 and 20 min, they were given an overview of the study objectives and procedures. They were then sent further information on the study including a participant information sheet (PIS) and informed consent form (ICF) via e-mail and offered the opportunity to ask further questions if needed.

Yoga teachers were recruited through an expression of interest sent by the studio hosting the yoga sessions. Following two rounds of interviews to ascertain the teachers’ certifications and teaching experience, as well as their availability and suitability for the study, the stakeholders were sent the stakeholders’ PIS and ICF via e-mail and offered the opportunity to ask further questions if needed.

#### Informed consent

Informed consent was obtained verbally and in writing at least 24 h after participants received the PIS and ICF. Stakeholder (yoga teachers) consent followed the same format.

### Inclusion criteria

#### Yoga participants

Inclusion criteria included being at or beyond week 20 of gestation, over 18 years old, having a basic understanding of English, being able to travel to the sessions, and self-identifying as belonging to an ethnic minority group. Exclusion criteria encompassed any physical or mental health diagnosis that would prevent them from attending yoga sessions as advised by the participant’s healthcare professional.

#### Stakeholders (yoga teachers)

Stakeholders (yoga teachers) were required to be over 18 years of age, to be of ethnic minority background themselves, fully certified (at least 200 h under a Yoga-Alliance program, an industry standard), have at least a year of yoga teaching experience and had to be insured to teach pregnant students.

#### Compensation

Participants were not financially compensated for their involvement in the study. However, after week 8 (end of intervention), each participant was provided with a yoga mat to encourage continued practice post-study. Conversely, the yoga teachers who facilitated the sessions were remunerated for their professional services at an industry-standard hourly rate.

### Data analysis

#### Quantitative analysis

Data collection was conducted using the Research Electronic Data Capture (REDCap) platform, and statistical analyses were performed using IBM SPSS Statistics version 28.0.1.1. An intention-to-treat approach was applied throughout the analysis. Baseline participant characteristics were summarized using descriptive statistics, including means, standard deviations (SD), and standard errors of the mean (SEM), while frequency analyses were used to examine the distribution of categorical variables.

Data were analyzed at two time points: an interim analysis at 4 weeks to explore attendance trends and early changes in clinical outcomes, and a final analysis at 8 weeks to comprehensively evaluate the intervention’s feasibility and impact. Repeated measures analyses were conducted for subjective outcomes assessed at baseline, 4 weeks, and 8 weeks, using general linear models for each outcome, with significance set at *p* < 0.05. Feasibility measures, including the Intervention Appropriateness Measure (IAM) and Feasibility of Intervention Measure (FIM), were analyzed at 8 weeks using frequency analyses, with results summarized through means, SDs, and SEMs to describe distribution and variability. Confidence intervals (CIs) were reported at a 95% confidence level.

#### Qualitative analysis

Qualitative data were gathered through focus groups and interviews, providing in-depth insights into participants’ experiences and perceptions. We employed a coding reliability thematic analysis, where three researchers independently coded the qualitative data on NVivo v12 [40]. As a methodological foundation, the six-step thematic analysis approach by Braun and Clarke [41] guided the analysis. It involved data familiarization, initial code creation, text coding, aggregating codes with supporting data, grouping codes into themes, and evaluating and revising themes and codes. Three independent researchers (CE, CC, and WK) conducted the coding process, ensuring inter-rater reliability. Discrepancies were resolved through discussion, leading to a consensus on the final codes and themes.

## Results

### Demographics

#### Participants

**Fig. 2:**
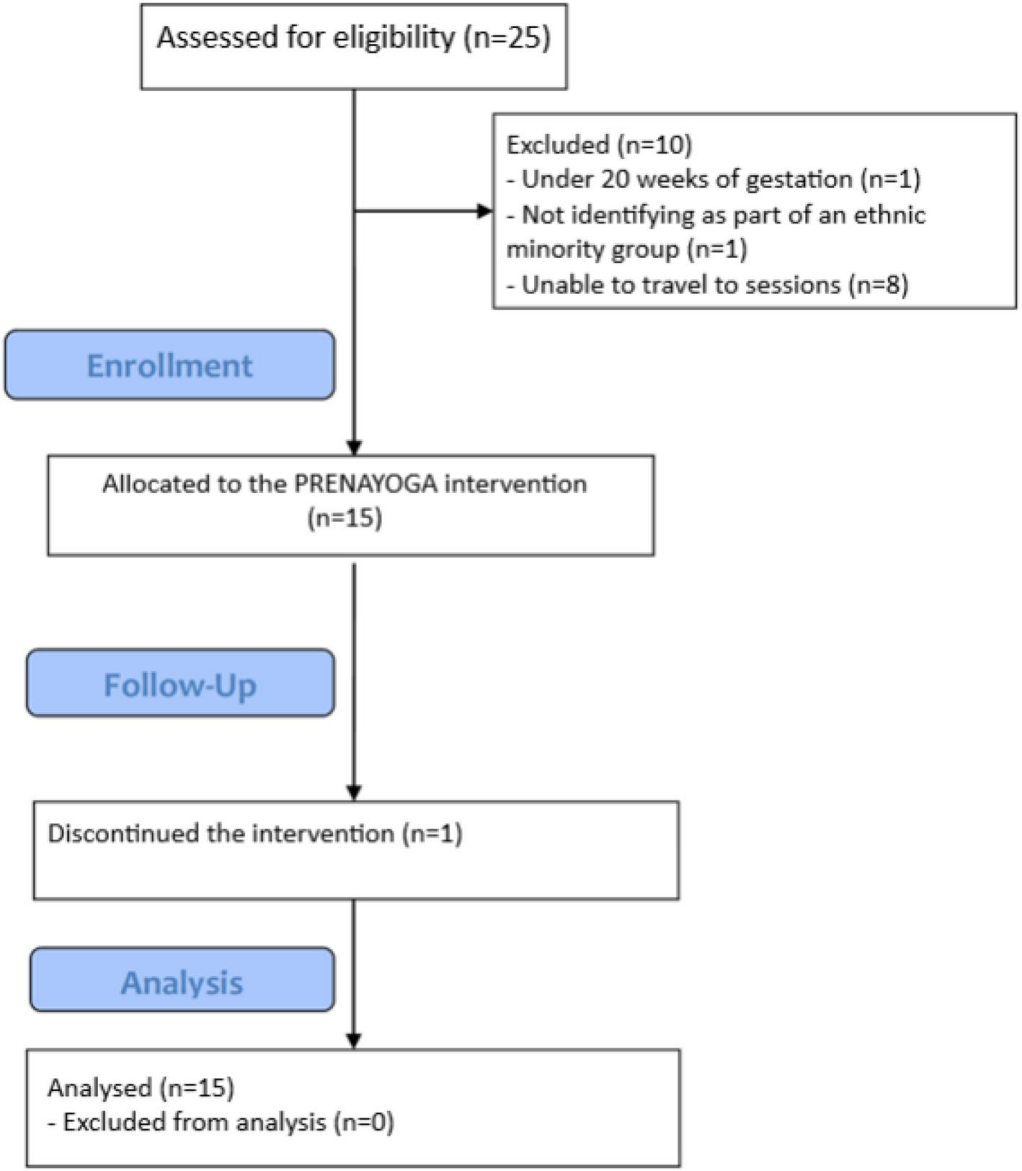
CONSORT diagram of participant flow through the PRENAYOGA study

A total of 25 potential participants completed the online screening, of which 10 were ineligible: under 20 weeks of gestation (*n* = 1), not identifying as part of an ethnic minority group (*n* = 1) or unable to travel to sessions (*n* = 8) (see Fig. 2). A total of 15 participants were enrolled in PRENAYOGA.

Participants, on average, were 33 years of age, and their gestational age was 25 weeks at baseline. Participants were of Caribbean (33.4%), Asian (26.6%), African (20%), and other (20%) descent. They were typically university-educated (73.3%), with combined household incomes of £45,000 per year on average (London average = £37,900 (42)) and in romantic partnerships (100%) (Table 5).

**Table 5:** Participant demographics

Participants characteristics (*N* = 15)	% Frequency (*N*)/mean (SD)
Age	33 (4.66)
Gestational age at baseline (weeks)	25 (3.79)
Ethnicity	
Caribbean	33.4% (5)
African	20% (3)
Mixed other	20% (3)
South Asian	13.3% (2)
Other Asian	13.3% (2)
Education	
Vocational/college (B.Tecs/NVQs etc.)	13.3% (2)
A-levels	6.7% (1)
Higher education(degree/diploma)	73.3% (11)
Missing	6.7% (1)
Marital status	
Single (with a partner)	40% (6)
Cohabiting	20% (3)
Married	40% (6)
Income	
£10,001 to £20,000	20% (3)
£20,001 to £30,000	20% (3)
£30,001 to £40,000	20% (3)
£60,001 to £70,000	13.3% (2)
£70,001 to £80,000	13.3% (2)
£80,001 +	13.3% (2)

Most participants (66.7%) experienced pregnancy-related issues, such as backache, fatigue, and nausea. The majority (66.7%) had other preexisting medical conditions, including mental health issues (26.7%), such as anxiety, mood disorders, eating disorders, and personality disorders (Table 12).

Most participants had previous yoga experience (60%) and were recruited primarily through Instagram (Table 13).

### Stakeholders (yoga teachers)

The two PRENAYOGA teachers had an average age of 32 years. One identified as African and the other as mixed Caribbean. On average, they had 1.5 years of experience teaching yoga (Table 14 in Appendix VI).

### Implementation

#### Qualitative findings: focus group and interviews

##### Participants

Participants attended a focus group at week 8 (*n* = 9). To explore the views of low-engaging participants, we also interviewed participants who discontinued the intervention (*n* = 1) or attended less than 50% of the sessions (*n* = 2); the findings are included in the narrative below.

Five themes emerged from the qualitative analysis of the focus group and interviews: (1) experience of yoga, (2) intervention impact outside of the yoga sessions, (3) facilitators for joining and attending, (4) attendance barriers, and (5) future research (for selected quotes see Appendix III).

###### Experience of yoga

Participants had positive experiences during the yoga classes, expressing feelings of enjoyment and community. While some participants found certain yoga poses challenging, they appreciated the overall sense of fulfillment from the intervention. Expectations of the yoga intervention were largely met, even among those with limited prior yoga experience. Many participants reported perceived physical health benefits, such as increased flexibility and relief from pregnancy-related issues. They were receptive to the different yoga poses (*asanas*) and appreciated their adaptations for different ability levels. Additionally, participants experienced mental health benefits, including relaxation and stress reduction. Yoga counseling, particularly the use of affirmations or mantras, was well-received and had a calming effect on participants.

Participants unanimously described the sessions as a “safe space” where they felt secure and free from judgment. They appreciated the absence of any feeling of being “the odd one out” and the supportive atmosphere. Some participants noted that the class fostered a sense of community, where they enjoyed each other’s company and shared the experience of their pregnancy journeys, leading to a heightened comfort level among attendees.

Participants generally found the studio environment pleasant but noted issues such as overcrowding, dampness, and noise from the train above; however, they valued not having to bring their yoga equipment. Additionally, participants gave positive feedback on the sessions’ frequency, length, and duration.

###### Intervention impact outside of yoga sessions

Most participants mentioned practicing yoga techniques (*asanas, pranayama*, and yogic counseling/*mantras*) at home, indicating a meaningful integration of yoga into their daily lives. Surprisingly, some participants received advice from midwives for their breastfed babies that aligned with the practices they learned in the yoga classes.

###### Facilitators for joining and attending

Participants identified several positive factors that motivated their enrolment and regular attendance. Some had prior interest or experience in yoga, which facilitated their decision to join the study. The availability of free classes was a significant incentive for attendance, especially considering the high costs of yoga classes in their area. The convenience of the studio’s location was another encouraging factor contributing to regular attendance.

Participants appreciated the opportunity for physical activity and a break from their daily routines. A strong sense of community and the chance to connect with other pregnant women was attractive. Indeed, the focus on ethnic minority-specific classes made PRENAYOGA particularly appealing to participants. Furthermore, one participant in the focus group highlighted feeling comforted by being taught by a minority teacher.

###### Attendance barriers

Some participants faced obstacles that resulted in missed sessions. Physical health issues, such as hip dysplasia and sciatica, prevented some individuals from attending regularly. Preexisting commitments, such as work, social events, or religious celebrations (Ramadan and Eid) and caring responsibilities also interfered with attendance.

###### Module adaptations

Participants would prefer an intervention earlier in pregnancy to become more proficient with the yoga techniques. Most participants favored in-person classes but saw value in virtual options for future interventions.

###### Future research

Participants expressed their interest in future research, indicating their willingness to engage in similar interventions. Participants suggested recruitment methods (Facebook groups, midwives, and pregnancy-related apps), more manageable questionnaires to reduce survey fatigue, and expressed willingness to provide biological samples (saliva and hair samples). The randomized controlled trial design received mixed responses, with some open to it and others believing that if they were not guaranteed yoga, they would not join a study.

##### Stakeholders

The stakeholders provided feedback on (1) preparation and transport to class, (2) teaching experience, (3) creating community, (4) module adaptations, and (5) future research (for selected quotes see Appendix IV).

###### Preparation and transportation to class

On average, teachers spent 30 to 45 min on class preparation. Teachers spent 90-min training for the prenatal yoga module, including a refresher.

One of the teachers walked for 15 min or cycled for 6 min, while the other had a longer commute by bus, ranging from 40 to 50 min.

###### Experience of teaching

Teacher #1 expressed a sense of openness and a lack of specific expectations before starting, describing the experience as a “beautiful surprise.” Teaching felt fulfilling, organic, and natural to her. She was particularly inspired by the group’s ability to share and create a safe space for themselves and acknowledge the diversity within the group.

Teacher #2 anticipated that the yoga sessions would be “lovely” and believed they would attract individuals looking to move their bodies and find a sense of community. She described feeling honored to be part of the experience.

###### Creating community

Teacher #1 emphasized the importance of creating a community within the yoga classes. She encouraged participants to view the classes as an opportunity to build a lasting community. She believed that the shared experience of pregnancy and yoga would be something the participants could always return to in their lives.

Teacher #1 observed that the group engaged in conversations, shared knowledge, and formed connections. She recognized the power of this community building, which extended beyond the physical practice of yoga.

Teacher #2 also stressed the significance of sharing the experience with others who were going through a similar journey, especially as a minority group. She highlighted the rarity of such a space and the sense of community it fostered.

###### Module adaptations

Teacher #1 suggested starting the intervention earlier in pregnancy to allow participants to become more familiar with yoga practices. She believed that an earlier start would enable participants to continue their practice at home later in their pregnancies.

Both teachers agreed that biweekly 60-min classes were appropriate for the yoga intervention. They considered the 8-week duration sufficient for the group, given that many participants were nearing their due dates.

###### Fidelity of the intervention

Both teachers offered insights into their approaches to the module. They generally viewed the module as a valuable framework. Teacher #1 incorporated meditation to ground participants and adapted the class to match the group’s energy. She particularly emphasized using meditation in every class to establish a spiritual connection between the participants and their babies. Teacher #1 highlighted that she did more internal chanting due to the group’s dynamic and used yogic counseling sensitively for this group of individuals.

Teacher #2 followed some aspects of the module, especially the breathwork, but took a more flexible approach. She loosely based her classes on the module, making adaptations to suit the specific needs of the participants. Both teachers included specific poses to strengthen the hips and pelvis, considering the unique needs of pregnant women. A final yoga module alongside the adaptations suggested, can be found in Appendix VIII.

###### Future research

Both teachers found the studio environment suitable, although Teacher #1 adjusted the layout to create a more inclusive atmosphere.

Finally, both teachers strongly desired to teach yoga to pregnant women in future studies. They found the experience rewarding and were enthusiastic about supporting pregnant women on their yoga journeys.

#### Quantitative findings: acceptability, appropriateness, and feasibility

Participants found the intervention acceptable (AIM), appropriate (IAM), and feasible (FIM). Participants scored, on average, 4.95 (SEM = 0.03, 95% CI = 4.89 to 5) on the AIM subscale; 4.77 (SEM = 0.13, 95% CI = 4.5 to 5) on the IAM; and 4.75 (SEM = 0.11, 95% CI = 4.54 to 4.9) on the FIM sub-scales (see Table 11 in Appendix V).

#### Attendance and drop-out rates

Participants attended, on average, 55.4% of the sessions, with 79.8% attending at least once a week (Table 6). Only one participant discontinued the intervention due to physical health concerns, unrelated to the intervention. Their data were, however, included in the analysis.

**Table 6:** Yoga session attendance

Average # of participants per class	10 (range 4–12)
Twice-a-week average attendance	55.4% (12.5–100%)
Once-a-week average attendance	79.8%
Highest attendance week	Week 2 (73%)
Lowest attendance week	Week 6 (37%)

^Percentages represent attendance frequency^

#### Exploratory clinical findings

##### Mental health

**Fig. 3:**
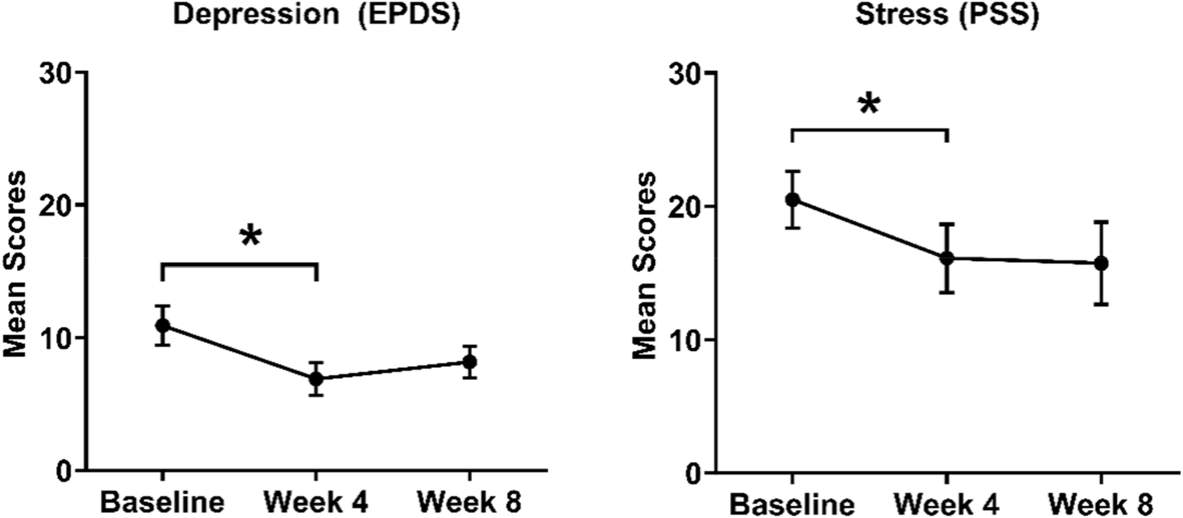
Mean EPDS and PSS scores at baseline, week 4, and week 8. Error bars show SEM *p < 0.05. EPDS, Edinburgh Postnatal Depression Scale; PSS, Perceived Stress Scale; SEM, standard error of the mean. * indicates *p* < 0.05

The intervention showed reductions in depressive symptoms, as measured by the EPDS at week 4 (Cohen’s *d* = 0.90, *p* = 0.02), indicating a large effect size; although the effect size slightly decreased by week 8 (Cohen’s *d* = 0.64, *p* = 0.06). Perceived stress, as measured by the PSS, decreased at week 4 (Cohen’s *d* = 0.55, *p* = 0.01), indicating reduction, but the effect was not statistically significant at week 8 (Cohen’s *d* = 0.55, *p* = 0.10). Overall, the intervention had a positive impact on reducing depressive symptoms and perceived stress, particularly in the short term (week 4), but the effects reduced over time (Fig. 3; also see Table 15 in Appendix VII).

Similarly, the BDI showed a reduction in depressive symptoms in week 4 (Cohen’s *d* = 0.81, *p* = 0.05), but the effect was not sustained in week 8 (Cohen’s *d* = 0.61, *p* = 0.17).

The intervention had moderate effects on reducing anxiety symptoms, with effect sizes ranging from 0.42 to 0.52 for state and trait anxiety. Specifically, state anxiety (STAI State) showed a moderate decrease at week 4 (Cohen’s *d* = 0.51) and week 8 (Cohen’s *d* = 0.52), although the reductions were not statistically significant (*p* = 0.23 and *p* = 0.27, respectively). Similarly, trait anxiety (STAI Trait) showed moderate decreases in week 4 (Cohen’s *d* = 0.43, *p* = 0.26) and week 8 (Cohen’s *d* = 0.42, *p* = 0.29), but the effects were not statistically significant. Overall, the intervention had some moderate effects on reducing anxiety symptoms, but they were not statistically significant (Figure 9; also see Table 15 in Appendix VII).

##### Quality of life

**Fig. 4:**
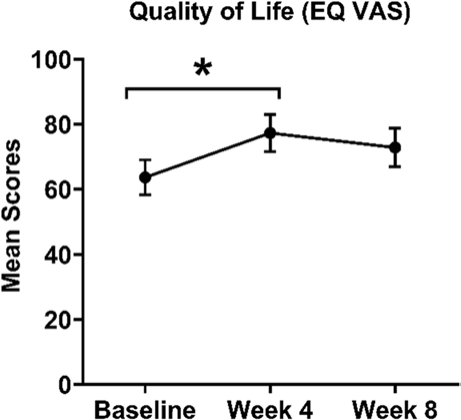
Mean EQ VAS scores at baseline, week 4, and week 8. Error bars show SEM. *p < 0.05. EQ-VAS, EuroQol Visual Analogue Scale; SEM, standard error of the mean

Quality of life (EQ-VAS score) improved from baseline to week 4 (*p* = 0.03) (Fig. 4). The yoga intervention had a large effect on the quality of life at week 4, as measured by EQ VAS (Cohen’s *d* = 0.85), but the effect size was moderate at week 8 (Cohen’s *d* = 0.52). In contrast, the effect sizes were smaller for the EQ5D-5L Index, with a small effect at week 4 (Cohen’s *d* = 0.22) and no effect at week 8 (Table 7). EQ5D-5L Index scores, measuring mobility, self-care, activity, pain, and anxiety levels, did not significantly improve throughout the intervention (Fig. 6 in Appendix VII).

**Table 7:** Quality of life

Questionnaire	Mean/(SD)	SEM	Effect size (Cohen’s *d*)	95% CI (Cohen’s *d*)	*p* (versus baseline)
EQ VAS Baseline Week 4 Week 8	63.58 (16.06) 77.27 (17.40) 72.80 (18.94)	5.40 5.79 5.99	0.85 0.52	(− 0.023, 1.723) (− 0.329, 1.369)	0.03* 0.23
EQ5D-5L Index baseline Week 4 Week 8	0.88 (0.094) 0.90 (0.086) 0.88 (0.098)	0.023 0.028 0.031	0.22	(− 0.619, 1.059)	0.55 0.17

*EQ-VAS EuroQol Visual Analogue Scale, *EQ-5D-5L* EuroQol 5-Dimension 5-Level index, *SD *standard deviation, *SEM *standard error of the mean, *CI *confidence interval, * indicates *p* < 0.05

##### Social support and self-efficacy

The intervention had small effects on social support (MSPSS) at week 4 (Cohen’s *d* = 0.18) and week 8 (Cohen’s *d* = 0.11) (in Appendix VII), and small effects on self-efficacy (GSE-6) at week 4 (Cohen’s *d* = 0.11) and week 8 (Cohen’s *d* = 0.19) (in Appendix VII). However, none of the changes was statistically significant, with *p*-values ranging from 0.47 to 0.87 for both MSPSS (*p* = 0.54 at week 4 and *p* = 0.58 at week 8) and GSE-6 (*p* = 0.87 at week 4 and *p* = 0.47 at week 8) (see Table 16 in Appendix VII).

##### Maternal–fetal attachment

The intervention had negligible effects on maternal prenatal attachment (MAAS and MFAS). Additionally, the intervention had small positive effects on prenatal attachment (PAI) at week 4 (Cohen’s *d* = 0.10) and week 8 (Cohen’s *d* = 0.21). However, none of the changes was statistically significant, with *p*-values ranging from 0.07 to 0.89 (see Table 17). However, absolute scores on the MFAS (Fig. 12 in Appendix VII) and PAI (Fig. 13 in Appendix VII) improved from baseline to week 8, unlike MAAS scores (Fig. 14 in Appendix VII). Overall, the intervention did not have a substantial impact on these outcomes.

**Fig. 5:**
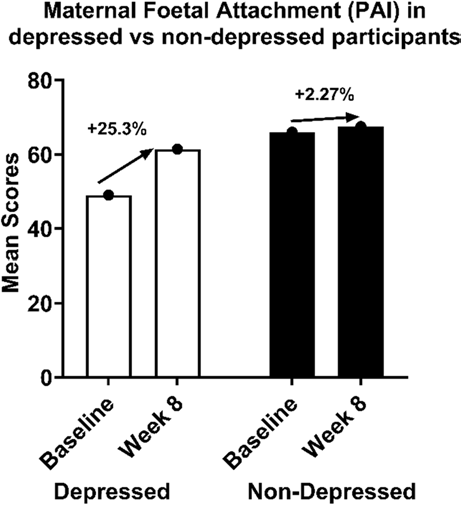
Attachment in depressed versus non-depressed participants (PAI) at baseline, week 4, and week 8 PAI, prenatal attachment inventory; SEM, standard error of the mean

To understand whether there were greater improvements in depressed participants, we conducted an additional analysis by dividing the sample into two subgroups, depressed (EPDS > 10) and non-depressed (EPDS < 10) at baseline. The depressed subgroup had lower attachment scores at baseline (PAI, *M* = 49). Despite not being significant, depressed participants experienced greater attachment improvements from baseline to week 8 (PAI + 25.30%), although this effect did not attain statistical significance (Fig. 5).

## Discussion

PRENAYOGA is the first study of its kind to examine the acceptability and feasibility of prenatal yoga for this pregnant women from ethnic minority backgrounds.

We found the PRENAYOGA intervention to be potentially acceptable, appropriate, and feasible for ethnic minority women in London, considering the study’s small sample size. This finding aligns with other studies [20, 24, 43] that also found prenatal yoga to be an acceptable intervention for pregnant women. In the qualitative analysis, a majority reported experiencing numerous physical and mental health advantages from participation. The intervention alleviated issues such as pelvic pain and enhanced flexibility, confirming previous reports in the literature [44, 45]. These observed benefits played a crucial role in promoting regular attendance, enhancing the feasibility of the intervention.

Our exploratory clinical findings indicate that prenatal yoga alleviated depressive symptoms and perceived stress and enhanced overall quality of life. There is also potential for prenatal yoga to improve attachment in prenatally depressed women. Though quantitative outcomes did not reach significance, qualitative feedback suggested perceived emotional connection improvements. Qualitative and quantitative data evidenced improvements in depressive symptoms and stress. These findings align with the literature on yoga’s mental health benefits [20, 24, 35, 43, 46]. Notably, participants who attended less than 50% still perceived similar physical and mental health benefits. These preliminary findings indicated potential reductions in depressive symptoms, though the study was not powered to detect clinical effectiveness.

Maternal–fetal attachment (MFA) reflects the cognitive, emotional, and physical representation of the relationship between the mother and fetus [47–49]. MFA behaviors increase progressively throughout pregnancy, accelerating with increasing fetal movement and gestational age [50]. From 28 gestational weeks, MFA increases significantly. We expected to find an increase from mid-intervention in MFA, which was not found in our sample. Overall, participants reported high baseline maternal–fetal attachment scores, which may have introduced a ceiling effect, reducing the likelihood of detecting significant changes across the intervention. However, we did however find a 25% increase in attachment following the 8-week intervention in women who were depressed as the baseline. Future studies may consider targeting individuals with lower baseline attachment scores or controlling for this in analysis.

Depressed individuals had lower attachment scores at baseline, as expected as per the literature on prenatal depression and maternal–fetal attachment [20, 45]. Interestingly, following the prenatal yoga intervention, the depressed subgroup showed the greatest improvement in attachment scores, indicating that prenatal yoga may enhance attachment in prenatal depression. This finding aligns with the existing research on the positive effect of prenatal yoga on attachment in prenatal depression and attachment-related benefits associated with prenatal yoga interventions [20, 45, 51]. The participants highlighted how they felt at ease, safe, and supported in a space with women looking like them. For once, they did not feel part of a minority but belonged to a group with a similar background. It emerged from the interviews that some of the participants had experienced racial trauma and discrimination. The prenatal yoga sessions in PRENAYOGA, an environment free from societal pressures, likely helped improve mental health outcomes. Additionally, both teachers expressed a sense of fulfillment and comfort in teaching this specific group of participants.

Both participants and Teacher #1 highlighted the importance of participants forming connections beyond yoga. Existing research confirms the importance of a supportive community during pregnancy [28]. Social support has a significant positive impact on maternal mental health during pregnancy and the postpartum period [28].

The analysis revealed that both teachers recognized the value of the provided module as a foundational framework. Nevertheless, they were flexible in their approach, modifying practices to align with the energy and requirements of the group. The consensus on the appropriateness of two 1-h sessions per week for an 8-week duration presents a viable model for future programs.

Even though traditional yoga classes do not include music, our classes included instrumental music since the teachers expected to teach the sound of music and it is culturally appropriate in London. Research has shown that both music and singing interventions during pregnancy can have immediate positive effects on emotional well-being and reduce stress [52]. Since we did not question individual’s experiences of the music, we do not know if some of the emotional benefits result from the music or the yogic practices.

PRENAYOGA encountered three limitations: a small sample size, drop-in attendance rates, and the absence of objective measures. The primary constraint is the small sample size, justified by the study’s exploratory feasibility focus, considering operational and ethical costs. Attendance declined by 54% after week 4, attributed to various factors such as social commitments and work. To address this, scheduling classes during periods with fewer conflicts or offering online sessions could improve attendance.

We did not record whether women were primipara or multipara. Primiparas tend to be less confident and feel more isolated [53]. We do not know if the measured benefits were greater due to women’s pregnancy experience. From a mechanistic viewpoint, the study lacked objective measures such as biological and neuroimaging markers. Given the nature of a feasibility study and the small sample size, it would not be possible to collect these samples; however, from the initial PPI group and the study’s focus group and interviews, participants were open to the collection of biological samples.

Additionally, from an acceptability perspective, given that 60% of participants had previous yoga experience, the intervention may have been more acceptable and less intimidating than for individuals unfamiliar with yoga. This prior exposure could have influenced both engagement levels and perceived benefits, and future studies should consider stratifying or controlling for baseline yoga familiarity.

Given the small sample size, it was not possible to adequately check the required assumptions of the statistical models employed. Consequently, the parameter estimates should be interpreted with caution, as they may be unreliable and subject to considerable sampling variability.

## Conclusion

The PRENAYOGA study aimed to assess the feasibility, acceptability, and potential for integrating a co-developed prenatal yoga intervention into perinatal care for ethnic minority populations. The feasibility benchmarks included acceptability, appropriateness, feasibility, attendance, attrition, and quality of life improvements. The intervention demonstrated high levels of acceptability, appropriateness, and feasibility, with AIM, IAM, and FIM scores exceeding the pre-specified threshold of 4 out of 5, indicating strong participant endorsement. Attendance benchmarks were nearly met, with 79.8% of participants attending at least once weekly, slightly below the 80% target, but consistent with other prenatal yoga interventions. Attrition was low (one dropout), meeting the ≤ 20% benchmark. While the study identified a trend towards improvement in quality of life at week 4, this effect was not sustained at week 8, potentially reflecting the natural decline in quality of life with gestational age.

Beyond feasibility benchmarks, qualitative findings strongly supported the acceptability and potential benefits of prenatal yoga for the target population. Participants highlighted a sense of community, cultural relevance, and a safe, non-judgmental space as key facilitators of engagement. Many integrated yoga techniques into daily life, reinforcing their perceived value beyond the sessions. Stakeholders emphasized the importance of cultural tailoring and suggested earlier interventions in pregnancy to enhance familiarity with the practice. While exploratory clinical findings indicated reductions in depressive symptoms and stress, alongside improved quality of life at week 4, the study was underpowered, and these trends must be interpreted cautiously. Maternal–fetal attachment showed no significant overall increase, though qualitative feedback suggested a stronger emotional connection to the pregnancy for some participants. Given the small sample size and feasibility focus, the study cannot determine clinical efficacy but highlights the potential for prenatal yoga to support mental well-being and social connectedness.

This study provides initial indications of potential benefits, although it was not designed or powered to detect definitive effects. As such, the observed trends warrant further investigation in larger, adequately powered studies.

Larger trials with sufficient power are necessary to validate the observed trends and further explore the scalability of the intervention. By focusing on the needs of pregnant women from ethnic minority backgrounds, the study contributes valuable insights to the field of perinatal care and lays the groundwork for broader implementation efforts.

## Supplementary Information

Supplementary Material 1

## Data Availability

The dataset(s) supporting the conclusions of this article is available upon request.
