# Supplementary material for: Co-designing and evaluating a prenatal yoga intervention for ethnic minority women: a feasibility study
Source: Pilot Feasibility Stud. 2025 Jul 7;11:96. doi: 10.1186/s40814-025-01667-9 (PMC12232825; doi:10.1186/s40814-025-01667-9)
Supplement: Supplementary file 1 — Supplementary Material 1 [file 40814_2025_1667_MOESM1_ESM.docx]

# Appendices

## Appendix I

Table 8 - Questionnaire for Experts

| Source | Type | Subtype | Name/Details | Strongly agree | Agree | Neither Agree or disagree | Disagree | Strongly disagree | Comments |
| --- | --- | --- | --- | --- | --- | --- | --- | --- | --- |
| I | Gestational Age |  | From second trimester of pregnancy |  |  |  |  |  |  |
| I | Schedule | Frequency | At least 3 times per week |  |  |  |  |  |  |
| I | Schedule | Size of class | Up to 15 participants per class |  |  |  |  |  |  |
| I | Schedule | Duration | Up to one hour |  |  |  |  |  |  |
| I | Schedule | Home practice | Simple loosening exercises and breathing practices |  |  |  |  |  |  |
| I | Schedule | Teleyoga | No classes delivered online for safety |  |  |  |  |  |  |
| I | Other | Soft ambient music during class | Instrumental music; same playlist the entire programme |  |  |  |  |  |  |
| I | Other | Themes (Y/N) | Weeks 1-3: introduction to asana, mindfulness meditation and breathing practices.  Weeks 4-6: introduction to home practices, introduction to chanting. Weeks 7-8: yoga for breech baby and yogic practices during labour |  |  |  |  |  |  |
| I | Sequence of the class |  | Loosening exercises, *asana* (standing), *asana* (sitting), *asana* (supine), *pranayama*, meditation. Throughout: counselling and chanting |  |  |  |  |  |  |
| I* | Asana | Loosening exercises (Preparation) | *sukhasana* rotations and side stretches |  |  |  |  |  |  |
| I* | Asana | Loosening exercises (Preparation) | neck exercise |  |  |  |  |  |  |
| I* | Asana | Loosening exercises (Preparation) | shoulder rotation |  |  |  |  |  |  |
| I* | Asana | Loosening exercises (Preparation) | elbow movement |  |  |  |  |  |  |
| I* | Asana | Loosening exercises (Preparation) | wrist rotation |  |  |  |  |  |  |
| I* | Asana | Loosening exercises (Preparation) | loosening of fingers |  |  |  |  |  |  |
| I* | Asana | Loosening exercises (Preparation) | waist rotation |  |  |  |  |  |  |
| I* | Asana | Loosening exercises (Preparation) | knee rotation |  |  |  |  |  |  |
| I* | Asana | Loosening exercises (Preparation) | ankle rotation |  |  |  |  |  |  |
| R | Asana | Loosening exercises (Preparation) | parivrtta sukhasana |  |  |  |  |  |  |
| R/I | Asana | Loosening exercises (Preparation) | *bitilasana*/marjariasana/tiger breathing/*vyaghrah pranayama* |  |  |  |  |  |  |
| R | Asana | Loosening exercises (Preparation) | bitilasana |  |  |  |  |  |  |
| R | Asana | Loosening exercises (Preparation) | *bitilasana* (bird-dog) |  |  |  |  |  |  |
| R | Asana | Standing and balancing poses | tadasana I |  |  |  |  |  |  |
| R/I | Asana | Standing and balancing poses | trikonasana |  |  |  |  |  |  |
| R/I | Asana | Standing and balancing poses | virabhadrasana I |  |  |  |  |  |  |
| R/I | Asana | Standing and balancing poses | virabhadrasana II |  |  |  |  |  |  |
| R/I | Asana | Standing and balancing poses | kneeling virabhadrasana |  |  |  |  |  |  |
| R | Asana | Standing and balancing poses | saaras pakshiasana |  |  |  |  |  |  |
| R/I | Asana | Standing and balancing poses | vrksasana |  |  |  |  |  |  |
| R/I | Asana | Standing and balancing poses | ardhakati chakrasana |  |  |  |  |  |  |
| I | Asana | Standing and balancing poses | prasarita padaottanasana |  |  |  |  |  |  |
| I | Asana | Standing and balancing poses | utkatasana |  |  |  |  |  |  |
| I | Asana | Sitting and keenling | parvatasana |  |  |  |  |  |  |
| R | Asana | Sitting and keenling | sukhasana |  |  |  |  |  |  |
| R | Asana | Sitting and keenling | garudasana |  |  |  |  |  |  |
| R/I | Asana | Sitting and keenling | baddha konasana |  |  |  |  |  |  |
| R/I | Asana | Sitting and keenling | upavistha konasana |  |  |  |  |  |  |
| R | Asana | Sitting and keenling | vajrasana |  |  |  |  |  |  |
| R | Asana | Sitting and keenling | gomukasana |  |  |  |  |  |  |
| R/I | Asana | Sitting and keenling | malasana |  |  |  |  |  |  |
| R | Asana | Sitting and keenling | siddhasana |  |  |  |  |  |  |
| R/I | Asana | Sitting and keenling | vakrasana |  |  |  |  |  |  |
| I | Asana | Sitting and keenling | ardha ustrasana |  |  |  |  |  |  |
| I | Asana | Sitting and keenling | titiliasana |  |  |  |  |  |  |
| I | Asana | Sitting and keenling | chakki chalanasana |  |  |  |  |  |  |
| I | Asana | Supine | uttanpadasana |  |  |  |  |  |  |
| I | Asana | Supine | setu bandhasana |  |  |  |  |  |  |
| R | Asana | Supine | ardha pavanamuktasana |  |  |  |  |  |  |
| R | Asana | Supine | jathara parivartanasana |  |  |  |  |  |  |
| I | Asana | Supine | matsyasana |  |  |  |  |  |  |
| I | Asana | Restorative | viparita karani |  |  |  |  |  |  |
| I | Asana | Restorative | parsva savasana |  |  |  |  |  |  |
| I | Pranayama |  | kapalbhati |  |  |  |  |  |  |
| I | Pranayama |  | bhramari |  |  |  |  |  |  |
| I | Pranayama |  | nadi shodhana |  |  |  |  |  |  |
| I | Pranayama |  | surya anuloma viloma |  |  |  |  |  |  |
| I | Pranayama |  | ujjayi |  |  |  |  |  |  |
| I | Pranayama |  | vibhagiya pranayama |  |  |  |  |  |  |
| I | Pranayama |  | sheetali pranayama |  |  |  |  |  |  |
| I | Meditation | Sound meditation | nadanusandhana |  |  |  |  |  |  |
| I | Meditation | Mindfulness | mindfulness meditation on breath and sensations |  |  |  |  |  |  |
| I | Meditation | Chanting | *japa* meditation |  |  |  |  |  |  |
| I | Meditation | Chanting | A-U-M chanting, followed by AUM chanting |  |  |  |  |  |  |
| I | Meditation | Chanting | *pranav* meditation |  |  |  |  |  |  |
| I | Meditation | Guided visualisation | Visualisation of baby-related themes |  |  |  |  |  |  |
| I | Meditation | Guided visualisation | body scan |  |  |  |  |  |  |
| I | Meditation | Deep relaxation meditation | yoga *nidra* (in parsva savasana or mandukasana) |  |  |  |  |  |  |
| I | Other/Counselling | 4 Yoga streams to deal with adversity | 4 yoga streams (jnana, bhakti, raja and karma). |  |  |  |  |  |  |
| I | Other/Counselling | Yogic anatomy model | *panchakosha* model (5 layers of existence) |  |  |  |  |  |  |
| I | Other/Counselling | Connection | connection exercises for mother-baby |  |  |  |  |  |  |
| I | Other/Counselling | Affirmations | affirmations during class: "“I trust my body”, “I will have a healthy and happy baby”, "I accept things as they are, I open my arms to what life brings”, “my baby is going to be a great human being”, “my baby is going to be strong, content, collected”, “you have prepared for this”, “you’ve done your best”, “trust your body; your body knows what to do”, “surrender to what’s to come, you are prepared to handle it” |  |  |  |  |  |  |
| I | Other/Counselling | Gratitude | *Namaskara* *mudra* during counselling to offer gratitude for their bodies, their babies, the time they've taken to do yoga |  |  |  |  |  |  |
| I | Other/Counselling | Chitta Vrittis | script on *pramana* (correct knowledge), *viparyaya* (misconception), *vikalpa* (conceptualisation), *nidra* (sleep), *smriti* (memory) |  |  |  |  |  |  |
| I | Other/Counselling | 4 Virtues for mental peace | script on the concepts of *maitri* (friendship), *karuna* (compassion), *mudit* (pure joy)*a*, and *upeksha* (indifference) |  |  |  |  |  |  |
| I | Other/Counselling | Limbs of Patanjali Yoga | yamas and niyamas |  |  |  |  |  |  |
| I | Other/Educational | Breech baby techniques | tiger breathing (*simhasana*), *badha konasana, adho mukha savanasa* walks, in *bitilasana* use music/light to guide the baby down |  |  |  |  |  |  |
| I | Other/Educational | Yoga for labour | *badha konasana* and exhale during contractions; between contractions practice relaxation techniques. Breathe using ujjayi pranayama to lengthen and depend breaths. Affirmations: "I a m dilating", "the birth is progressing", "the baby is ready to arrive". Move around between contractions |  |  |  |  |  |  |
| \| R - literature review \| \| --- \| \| I* - from interview, added from book \| \| I - Interviews \| \| Note: prone and forward folding asanas were removed due safety and comfort concerns for the participants. \| | | | | | | | | | |

## Appendix II

- 1. **Interview Topic Guide (Focus Group and Interview) for Participants**

Introducing yourself:

Hello, my name is [Your Name], and I am [Your Role] in this study. I am here to learn about your experiences with the prenatal yoga programme you participated in. Your insights will help us refine the programme to better suit your preferences and those of other women with similar backgrounds.

- Purpose: We are seeking your experiences to refine the prenatal yoga programme for future participants.
- Voluntary Participation:
  - You have the right to skip any questions you do not wish to answer.
  - You may ask for a break at any point.
  - If you wish to stop the group or withdraw from the study for any reason, please let me know.
- Confidentiality:
  - I will be audio recording our conversation to create transcriptions.
  - Once transcripts are completed, audio recordings will be destroyed to ensure your confidentiality and anonymity.

1. Experience of the Intervention During Pregnancy (20 minutes):
   1. Expectations:
   - Was the programme what you expected before you started the study?
   1. Favourite Moments:
   - What were your favourite moments of the sessions? Why?
     - Were there specific poses you enjoyed?
     - Was the pace fast, just right, or too slow?
     - How did you find the breathwork?
     - What was your experience with meditation?
     - Did you experience any injuries from the practice?
   1. Disliked Moments:
   - Were there moments you disliked in the sessions? Why?
   1. Emotional and Physical Impact:
   - How did the yoga make you feel?
     - Did you notice any changes in your mood, emotions, feelings, or physical state?
   1. Home Practice:
   - Did you practice at home on your own?
2. Questionnaire Acceptability:
   - Were the questionnaires acceptable to you?
     - Demographics
     - Social support
     - Mental health
     - Attachment
3. Implementation – How to Make This Work in the Real World (20 minutes):
   1. Facilitators and Barriers to Attendance:
   - What motivated you to attend and enjoy the sessions?
   - What obstacles did you encounter in attending and enjoying the sessions?
     - If you missed sessions, what were the reasons? (e.g., religious festivities, bank holidays, weekends, school holidays)
   1. Perceived Acceptability of the Intervention:
   - What aspects of the session made you return each week?
   1. Appropriateness of the Intervention for Pregnancy:
   - What are your thoughts on the length of sessions, types of poses, meditation, chanting, and breathing exercises?
   - Did you like them? Were you comfortable? If not, why?
   1. Feasibility of Attending Sessions:
   - Were the sessions too frequent or not frequent enough?
   - Was the timing of the sessions suitable (e.g., were Wednesday sessions too late or were weekend sessions unsuitable)?
   1. Factors Affecting Sustainability and Scalability:
   - What factors do you perceive as affecting the sustainability and scalability of the intervention in the longer term (e.g., recruitment, acceptability by other pregnant women)?
   - Were the recruitment methods appropriate (e.g., Facebook groups, Instagram ads, posters in yoga studios and cafes, Instagram posts by other teachers)?
   - How did you find out about the study and sign up?
   - Do you think others would sign up if they saw the poster, or did you sign up because you already wanted to or had previously done yoga?
   - Would people with similar backgrounds find the recruitment appealing?
   - Do you think others with similar backgrounds would enjoy prenatal yoga?
4. Future Research (10 minutes):

We aim to conduct a larger study in the future and would like your opinion on some aspects. Please consider this as if you were pregnant (as you are now) and were joining a new study.

- 1. Willingness to Undergo Randomisation in an RCT:
  - Would you be willing to be randomly allocated to a group where there is a chance you may not receive the yoga intervention, or would you participate only if the yoga intervention was guaranteed?
  1. Preference for Session Format:
  - Do you prefer face-to-face sessions, synchronous online sessions (live online classes), or a hybrid format?
  1. Acceptability of Saliva Sample Collection:
  - Saliva samples would assess stress, bonding, and inflammation; no genetic analysis would be conducted, data would be anonymous, and samples destroyed after analysis.
    - Would you be open to providing saliva samples at home and during sessions?
  1. Acceptability of Hair Sample Collection:
  - Hair samples assess stress hormone output over a 3-month period; hair would be cut, not pulled, ensuring no genetic information is obtained.
    - Would you be open to providing hair samples?

1. Closing (5min). That’s all my questions complete. Thank you for answering my questions. I will now stop the audio recording.
   - What were your thoughts on the interview? How did you find the questions? Was this comfortable for you?
   - Do you have any further questions?

If you have any second thoughts about your participation in the study and wish to withdraw your data from the study, please let me know within the next two weeks. Should you have any further questions, please feel free to get in touch. If you feel that you need support, please see participant information sheet that was emailed to you at the start of the study which contains information on support services. Thank you for participating!

- 1. **Interview Topic Guide for Participants with Less Than 50% Attendance or Withdrawal**

Introducing yourself:

Hello, my name is [Your Name], and I am [Your Role] in this study. I am here to learn about your experiences with the prenatal yoga programme you participated in. Your insights will help us refine the programme to better suit your preferences and those of other women with similar backgrounds.

- Purpose: We are seeking your experiences to refine the prenatal yoga programme for future participants.
- Voluntary Participation:
  - You have the right to skip any questions you do not wish to answer.
  - You may ask for a break at any point.
  - If you wish to stop the group or withdraw from the study for any reason, please let me know.
- Confidentiality:
  - I will be audio recording our conversation to create transcriptions.
  - Once transcripts are completed, audio recordings will be destroyed to ensure your confidentiality and anonymity.

1. Initial Interest in the Study

- Discovery of the Study: How did you first learn about this study?
- Motivations for Participation:
  - What aspects of the study or its promotional materials encouraged you to sign up?
  - Were you interested in meeting other pregnant women, had you heard about pregnancy yoga before, or were you seeking prenatal education?

1. Reasons for Discontinuing Participation

- Attendance Challenges:
  - What factors contributed to your attending fewer than 50% of the sessions or withdrawing from the study?
  - Did any of the following affect your ability to attend the sessions?
    - Session timings
    - Duration
    - Distance from home
    - Childcare needs
    - Physical discomfort
  - Expectations vs. Reality:
    - Did the sessions meet your expectations?
    - Were there specific aspects, such as the poses, the instructor, class length, or physical demands, that influenced your decision to discontinue?

1. Overall Perspectives on the Intervention
   - Were there elements of the sessions that you enjoyed?
   - If you were to start again, would you change anything about the sessions?
   - Would you prefer if the classes were delivered virtually or in a hybrid model (some in person, some online)?
2. Feedback on Questionnaires
   - Did you think the questions in the online questionnaires were suitable?
   - Did you find the number of the questionnaires acceptable?
   - Did you find the questions relevant?
   - Did we miss any questions that you think we should have asked?
3. Acceptability of Biological Sample Collections
   - In a future study, would you be willing to provide saliva and hair samples? These would be used to analyse stress hormones (cortisol), bonding with the baby, and inflammation in the body. Please note, no genetic testing would be conducted.
4. Interest in Randomisation for Future Research
   - Future Participation:
   - In a future study, would you be interested (imagine you are pregnant again) in being randomised to a group where you might be allocated to a prenatal education group or a prenatal yoga group?
5. Closing (5min). That’s all my questions complete. Thank you for answering my questions. I will now stop the audio recording.
   - What were your thoughts on the interview? How did you find the questions? Was this comfortable for you?
   - Do you have any further questions?

If you have any second thoughts about your participation in the study and wish to withdraw your data from the study, please let me know within the next two weeks. Should you have any further questions, please feel free to get in touch. If you feel that you need support, please see participant information sheet that was emailed to you at the start of the study which contains information on support services. Thank you for participating!

- 1. **Stakeholder Topic Guides – Interview**

Introducing yourself:

Hello, my name is [Your Name], and I am [Your Role] in this study. I am here to learn about your experiences teaching the prenatal yoga programme. Your insights will help us refine the programme to better suit your preferences and those of the participants in the class.

- Purpose: We are seeking your experiences to refine the prenatal yoga programme for future teachers and participants.
- Voluntary Participation:
  - You have the right to skip any questions you do not wish to answer.
  - You may ask for a break at any point.
  - If you wish to stop the group or withdraw from the study for any reason, please let me know.
- Confidentiality:
  - I will be audio recording our conversation to create transcriptions.
  - Once transcripts are completed, audio recordings will be destroyed to ensure your confidentiality and anonymity.

1. Experience of the Intervention in Pregnancy
   - How did you feel delivering the yoga sessions?
   - What did you think about the module that you were trained on?
   - How did you feel working with the study group?
2. Expectations vs. Reality
   - What did you expect it was going to be like before you started teaching the sessions?
   - Was it what you expected before you started the study?
3. Teaching Environment
   - Did you think the studio was appropriate for the sessions? Was all the equipment there? Was it comfortable?
4. Module Acceptability
   - What did you think of:
     - Types of poses
     - Meditation
     - Chanting
     - Breathing exercises
     - Yogic counselling
   - Adaptations and Omissions
     - Was there anything you left out from the module?
     - Was there anything you added to the sessions that weren’t in the module (e.g., music, other poses, breathing techniques, meditations)?
5. Implementation
   - If you were invited in the future to teach this programme again, is there anything that would make you not want to do it again?
   - Is there anything that would make you want to do it again?
   - What was it in the sessions that you think worked for the group?
   - Was it the setting that made the difference? The specifics of the group? The fact that it was free to attend? Was it you as a teacher?
6. Appropriateness for Pregnancy
   - Did you think the length of the classes was appropriate? Was it too short or too long?
   - Did you think the timing of the sessions was appropriate?
   - Did you think 8 weeks (bi-weekly) was appropriate for the group or should it have been shorter or longer?
7. Feasibility of Delivery
   - Was it feasible for you to deliver all the sessions (all 16)? If not, why? Which arrangements did you make?
8. Factors Affecting Sustainability and Scalability
   - Do you think this yoga programme can be delivered in the future? Do you think it’s appealing to teachers and students?
   - Do you think if this wasn’t a research programme, it would have more or less success (for ethnic minority people)?
9. Closing (5min). That’s all my questions complete. Thank you for answering my questions. I will now stop the audio recording.
   - What were your thoughts on the interview? How did you find the questions? Was this comfortable for you?
   - Do you have any further questions?

If you have any second thoughts about your participation in the study and wish to withdraw your data from the study, please let me know within the next two weeks. Should you have any further questions, please feel free to get in touch. Thank you for participating!

## Appendix III

Table 9 – Participants: Themes and sample quotes

| Theme | Sample Quotes |
| --- | --- |
| Experience of Yoga | “[…] after yoga class, I'm much more flexible, and I can even bend forward. I think that’s been very helpful.”  "I think it was perfect, it was brilliant. To be honest with you. It wasn't too strenuous, it stretched me, I did feel a lot better from it, and mentally and physically, from the sessions.”  “Like I remember the mantras ‘cause they are all about trusting yourself or knowing that everything, there was one that said like everything, ‘I’ve got everything I need within me to do this’ so that was quite calming for certain moments.”  “To start you think, you start looking at her [the teacher] like, ‘I can’t do that’ but then actually, it’s good ‘cause she’s giving you something challenging and you surprise yourself ‘cause you can actually do it.”  She’s always like checking in and we didn't have to ask for modifications, it was kind of like ‘I understand that you've got a belly so you’re a lot bigger than others so if this isn't going to work, then try this or only go as far as you can’, and you know it was going with like she's actually taken into account that we are all pregnant women of different stages of pregnancy.”  “So, that was nice that we didn't feel like, you know, we, I don't know, like nobody kind of felt like they were the odd one out, or people like looking at them, or laughing at them. It was just, it was a nice space, it was very safe, and I think it was very safe environment.”  “Little bit cramped at times when it was a full class like 'cause I would find that with some of the movements like this, I would bang into other people and yeah, so I think maybe we were a bit oversubscribed at times or not spread out enough“  “I feel like the two sessions a week were perfect as well. It was just enough” |
| Intervention Impact Outside of Yoga Sessions | “[my] midwife has given me like lots of exercises to do, and I’m like ‘oh, I know that from yoga "classes' so do you know what I mean. It’s like ‘I can do this’.” |
| Facilitators for Joining and Attending | “I could not afford to do it this time around, and so I thought, you know it’s [this study] a free, free sessions. So, I'll just give it a try this time round”.  “was in bed at lot and not very mobile so [she] thought it was good for [her] to get up and moving in and get [her] foot joints moving”  “[…] I kind of been a bit like, isolated to this pregnancy and so I didn't really know any, I didn't know anybody else that was, that was pregnant, and I thought, that was kind of attractive appealing to me as well.”  “Also I feel like having people, women should I say that, who look like me as well ‘cause I’ve been to yoga class when I’ve not been pregnant, and it’s just been like me in a room full of white women, sorry, but it’s kind of, it’s very intimidating and I’m the only black woman in the room… cause that was one of the main, I want to say barriers to going into yoga practise.”  “Yeah so, and the teacher. My first impression of her is she is Black, which was nice. She’s attentive. She’s professional. I’ve never had a teacher of colour actually, ever teach me yoga, I know there are some, I’m not saying there aren’t, but like that was also a comfort.” |
| Attendance Barriers | “I've just been like really, really uncomfortable and a lot of pain and I just thought, I don't want to exasperate it by going to the yoga.”  “She's [daughter] on the spectrum. basically, of autism. Her mood's really been quite bad. Saturday was really bad. I was really upset. I couldn't come. So, I had to be around.” |
| Future Research | “I personally prefer face-to-face, the in-person one but I do feel like having the option to do a virtual class would be, it would be positive. Like, there are days when you know, sometimes that you're pregnant, you're tired and having to go somewhere else after work, or having to get up and go somewhere, could be made easier then, by just being able to do it in your living room. I feel like the whole experience of having in-person and having that, just that energy and being around other people and then it encourages you, you know it’s kind of hard to encourage yourself when you're back home. But having the option to do actually, with both would be good.“ |

## Appendix IV

Table 10 – Stakeholders (yoga teachers): Themes and Sample Quotes

| Theme | Sample Quotes |
| --- | --- |
| Teaching Experience | “I think straight off the bat just felt very, I felt very comfortable, holding a space“.  “I felt great, you know. Oh, different characters, different, very diverse even, even though it's, you know, people of colour still very diverse, you know, different languages, different, you know, different cultures, different stages of pregnancy.” |
| Creating Community | “you know, you, you, it's ‘you guys will always have that amongst each other’ […] So, just use this as just the opportunity to get to know each other, you know and just create a community that is comfortable for you.”  “This is not just about the yoga, is it? It's about just sharing, sharing space, sharing knowledge outside of it as well, and the continuation of, of their journey, yeah.”  “it's so rare to get a space of all black and brown women or women of colour in one place, all practising yoga, all pregnant and you know.” |
| Module Adaptations | “[…] I found that with from Teacher #1 like what kind of breath, what they've been doing. […] I think we went to like box breath, ujjayi breath. Which was in the […] module guide [training booklet] anyway.”  “I did more internal chanting. Because of the, the dynamics of the group. Hum, people of colour aren't really inclined, they don't really. I think they, they find it harder to do that. It stems from religious, religious backgrounds. So, […] I would always do some like you know ‘in the mind's eye’, 'can you recite this, […], recite this mantra’ or, or just play the music, play the, the chanting and you just you know, for at least two minutes and then you sit there. So, it was it was still done but in a different, more sensitive way.“ |
| Future Research | “Like as close to as close to 20 weeks basically. And then practise, because when you get to this stage, when you get to when you're like, really uncomfortable you, you just do your own practise at home for 10 minutes.”  "Absolute would want to do that. I'll definitely want to do that again. I couldn't think of why not." |

##

## Appendix V

Table 11 - AIM, IAM and FIM scores

| Questionnaire | Mean | SD | SEM | DF |
| --- | --- | --- | --- | --- |
| AIM | 4.95 | 0.10 | 0.03 | 11 |
| IAM | 4.77 | 0.45 | 0.13 | 11 |
| FIM | 4.75 | 0.38 | 0.11 | 11 |

AIM = Acceptability of Intervention Measure; IAM = Intervention Appropriateness Measure; FIM = Feasibility of Intervention Measure; SD = Standard Deviation; SEM = Standard Error of the Mean.

## Appendix VI

Table 12 – Participants’ Clinical Characteristics

| Participants Characteristics (N = 15) | % Frequency (N) / M (SD) |
| --- | --- |
| Pre-existing Chronic Condition |  |
| No | 33.3% (5) |
| Yes | 66.7% (10) |
| Mental Health | 26.7% (4) |
| Anxiety Disorders | 13.3% (2) |
| Mood Disorders | 13.3% (2) |
| Eating Disorders | 6.7% (1) |
| Personality Disorders | 6.7% (1) |
| Physical Health |  |
| Gynaecological | 20% (3) |
| Respiratory | 13.3% (2) |
| Endocrine | 13.3% (2) |
| Other | 13.3% (2) |
| Skeletal | 6.7% (1) |
| Immunological | 6.7% (1) |
| Musculo | 6.7% (1) |
| Pregnancy Physical Problems |  |
| No | 33.3% (5) |
| Yes | 66.7% (10) |
| Backaches | 80% (12) |
| Nausea and vomiting or hyperemesis | 53.3% (8) |
| Tiredness or fatigue | 53.3% (8) |
| Headaches | 53.3% (8) |
| Pelvic, Hip, lower Abdomen Pain | 26.7% (4) |
| Gestational diabetes | 20% (3) |
| Sore or cracked nipples | 20% (3) |
| Hypertension | 13.3% (2) |
| Sciatica | 13.3% (2) |
| Haemorrhoids | 13.3% (2) |
| Red or tender breasts or mastitis | 13.3% (2) |
| Other | 13.3% (2) |
| Perineal pain | 6.7% (1) |
| Bowel problems | 6.7% (1) |
| Bladder problems | 6.7% (1) |
| Sexual problems | 6.7% (1) |

SD = Standard Deviation; M = Mean.

Table 13 – Recruitment and previous yoga experience

| Participants Characteristics (N = 15) | % Frequency (N) / Mean (SD) |
| --- | --- |
| Recruitment Channel |  |
| Instagram ad | 46.7% (7) |
| Missing | 33.3% (5) |
| Facebook groups | 6.7% (1) |
| Yoga studio | 6.7% (1) |
| Other | 6.7% (1) |
| Yoga Experience at Baseline (1hr in the past month) |  |
| No | 60% (9) |
| Yes | 40% (6) |

SD = Standard Deviation; M = Mean.

Table 14 – Stakeholders demographics

| Stakeholders (yoga teachers) N=2 | Mean (SD)/ % Frequency (N) |
| --- | --- |
| Age | 32 (6.5) |
| Ethnicity |  |
| African | 50% (1) |
| Mixed Caribbean | 50% (1) |
| Years of Yoga teaching experience | 1.5 (2) |

SD = Standard Deviation; M = Mean.

## Appendix VII

Quality of Life


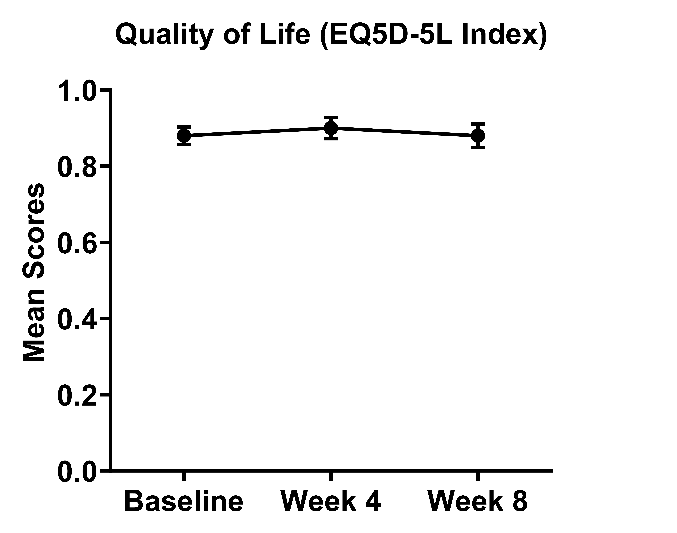


Figure 6 – Quality of life index (EQ-5D-5L mean scores and SEM at baseline, week 4 and week 8). EQ-5D-5L = EuroQol 5-Dimension 5-Level instrument; SEM = Standard Error of the Mean.

Mental Health Outcomes

Table 15 – Mental Health Outcomes

| Questionnaire | Mean/(SD) | SEM | Effect Size (Cohen’s d) | 95% CI (Cohen’s d) | p (versus baseline) |
| --- | --- | --- | --- | --- | --- |
| EPDS Baseline     Week 4     Week 8 | 10.92 (4.87)  6.90 (3.97)     8.18 (3.60) | 1.50  1.25  1.19 | 0.90  0.64 | (0.27, 1.53)  (0.05, 1.23) | 0.02*  0.06 |
| BDI Baseline     Week 4     Week 8 | 15.17 (9.17)    8.40 (7.36)    9.91 (8.15) | 2.81  2.58  3.02 | 0.81  0.61 | (0.14, 1.48)  (-0.03, 1.25) | 0.05  0.17 |
| STAI *State* Baseline     Week 4     Week 8  STAI *Trait* Baseline     Week 4     Week 8 | 46.54 (14.53)  39.64 (12.34)  38.35 (16.88)  43.00 (13.15)  37.45 (12.63)  37.25 (14.39) | 3.76  4.11  5.34  4.05  4.18  4.55 | 0.51  0.52  0.43  0.42 | (-0.05, 1.07)  (-0.05, 1.09)  (-0.11, 0.97)  (-0.11, 0.96) | 0.23  0.27  0.26  0.29 |
| PSS Baseline     Week 4     Week 8 | 20.50 (8.05)  16.10 (8.08)  15.73 (9.27) | 2.14  2.55  3.08 | 0.55  0.55 | (-0.02, 1.12)  (-0.04, 1.14) | 0.01 *  0.10 |

EPDS = Edinburgh Postnatal Depression Scale; BDI = Beck Depression Inventory; STAI = State-Trait Anxiety Inventory; SD = Standard Deviation; SEM = Standard Error of the Mean; * indicates p < 0.05.

Depression
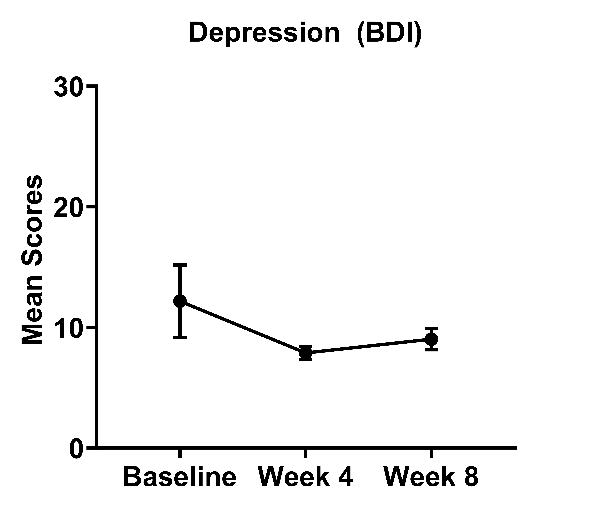


Figure 7 – Depression scores (BDI Mean and SEM at baseline, week 4 and week 8). BDI = Beck’s Depression Inventory; SEM = Standard Error of the Mean.

Anxiety


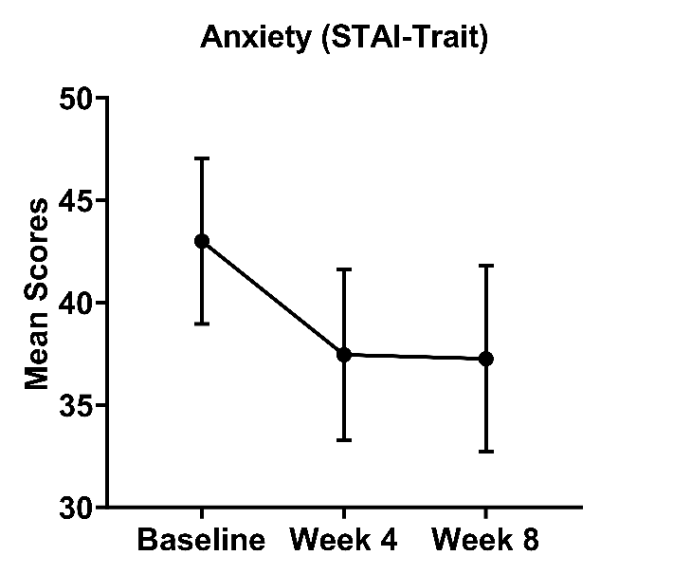


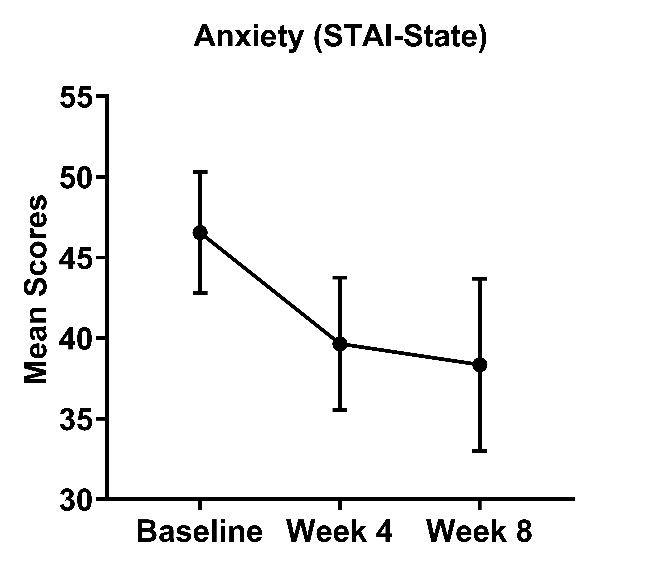
Figure 8 – Anxiety scores (STAI-State mean and SEM at baseline, week 4 and week 8). STAI-Trait= State-Trait Anxiety Inventory, trait subscale; SEM = Standard Error of the Mean.

Figure 9 – Anxiety scores (STAI-Trait mean and SEM at baseline, week 4 and week 8). STAI-State= State-Trait Anxiety Inventory, state subscale; SEM = Standard Error of the Mean.

Social Support and Self-Efficacy

Table 16 - Social Support and Self-efficacy Outcomes

| Questionnaire | Mean / (SD) | SEM | Effect Size (Cohen’s d) | 95% CI (Cohen’s d) | p (versus baseline) |
| --- | --- | --- | --- | --- | --- |
| MSPSS Baseline  Week 4  Week 8 | 71.83 (9.29)  73.36 (7.90)  72.90 (10.53) | 2.71  2.62  3.33 | 0.18  0.11 | (-0.45, 0.81)  (-0.51, 0.73) | 0.54  0.58 |
| GSE-6 Baseline  Week 4  Week 8 | 17.92 (3.47)  18.27 (3.13)  18.59 (3.62) | 0.78  1.14  1.26 | 0.11  0.19 | (-0.58, 0.80)  (-0.51, 0.89) | 0.87  0.47 |

MSPSS = Multidimensional Scale of Perceived Social Support; GSE-6 = General Self-Efficacy Scale (6-item version); SEM = Standard Error of the Mean.

Social Support


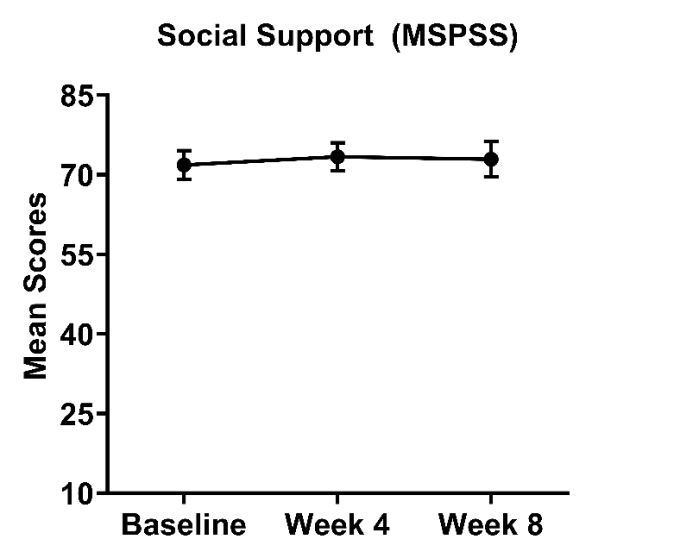


Figure 10 – Social support (MSPSS mean and SEM at baseline, week 4 and week 8). MSPSS = Multidimensional Scale of Perceived Social Support; SEM = Standard Error of the Mean.


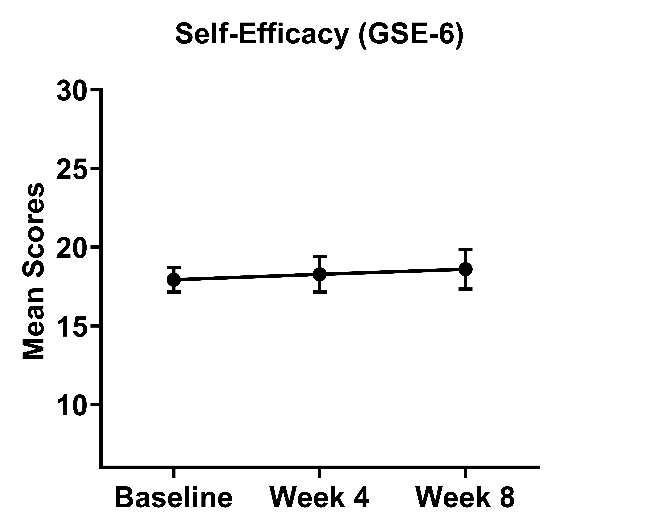
Self-Efficacy

Figure 11 – Self-Efficacy (GSE-6 Mean and SEM at baseline, week 4 and week 8). GSE-6 = General Self-Efficacy Scale (6-item version); SEM = Standard Error of the Mean.

Attachment

Table 17 - Attachment Outcomes

| Questionnaire | Mean / (SD) | SEM | Effect Size (Cohen’s d) | 95% CI (Cohen's d) | p (versus baseline) |
| --- | --- | --- | --- | --- | --- |
| MAAS Baseline  Week 4  Week 8 | 49.31 (3.54)  49.33 (2.38)  48.55 (2.25) | 0.84  0.75  0.67 |  |  | 0.86  0.45 |
|  |  |  | 0.007  -0.26 | (-0.56, 0.57)  (-0.84, 0.32) |  |
| MFAS  Baseline  Week 4  Week 8 | 91.54 (16.32)  90.17 (13.65)  93.27 (14.58) | 4.57  4.20  4.39 |  |  |  |
|  |  |  | -0.09  0.11 | (-0.66, 0.48)  (-0.46, 0.68) | 0.89  0.12 |
| PAI State  Baseline  Week 4  Week 8 | 62.08 (13.38)  63.36 (12.54)  64.64 (11.07) | 4.05  4.11  3.66 |  |  |  |
|  |  |  | 0.10  0.21 | (-0.53, 0.73)  (-0.43, 0.85) | 0.19  0.07 |

MAAS = Maternal Antenatal Attachment Scale; MFAS = Maternal Foetal Attachment Scale; PAI = Prenatal Attachment Inventory; SD = Standard Deviation; SEM = Standard Error of the Mean; CI = Confidence Interval; * indicates p < 0.05.


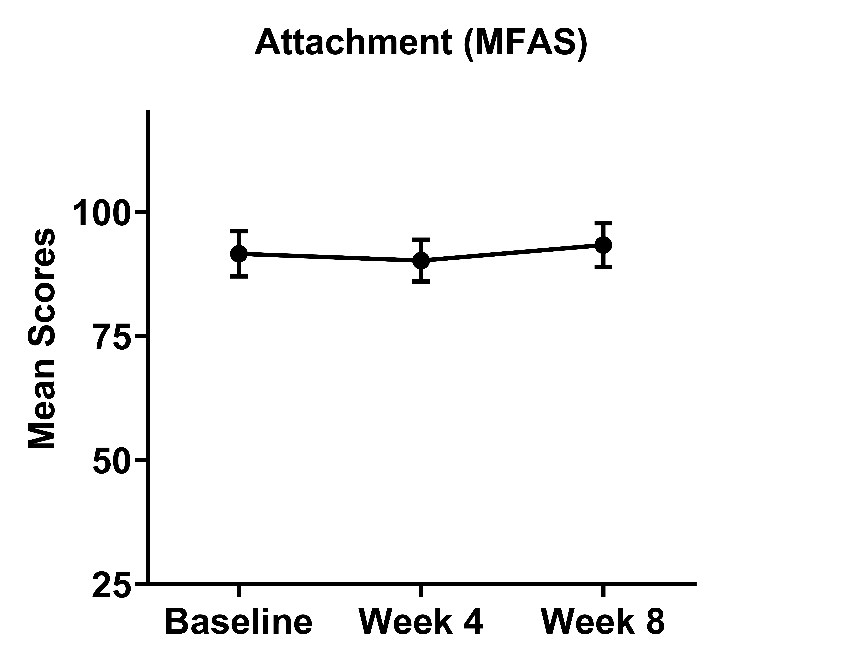


Figure 12 – Maternal foetal attachment (MFAS Mean and SEM at baseline, week 4 and week 8). MFAS = Maternal Foetal Attachment Scale; SEM = Standard Error of the Mean.


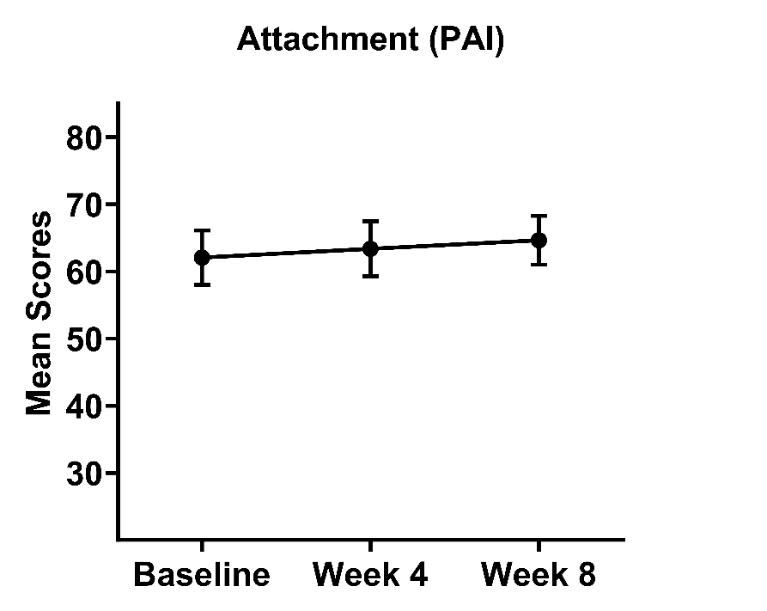


Figure 13 – Prenatal attachment inventory (PAI Mean and SEM at baseline, week 4 and week 8). PAI = Prenatal Attachment Inventory; SEM = Standard Error of the Mean.


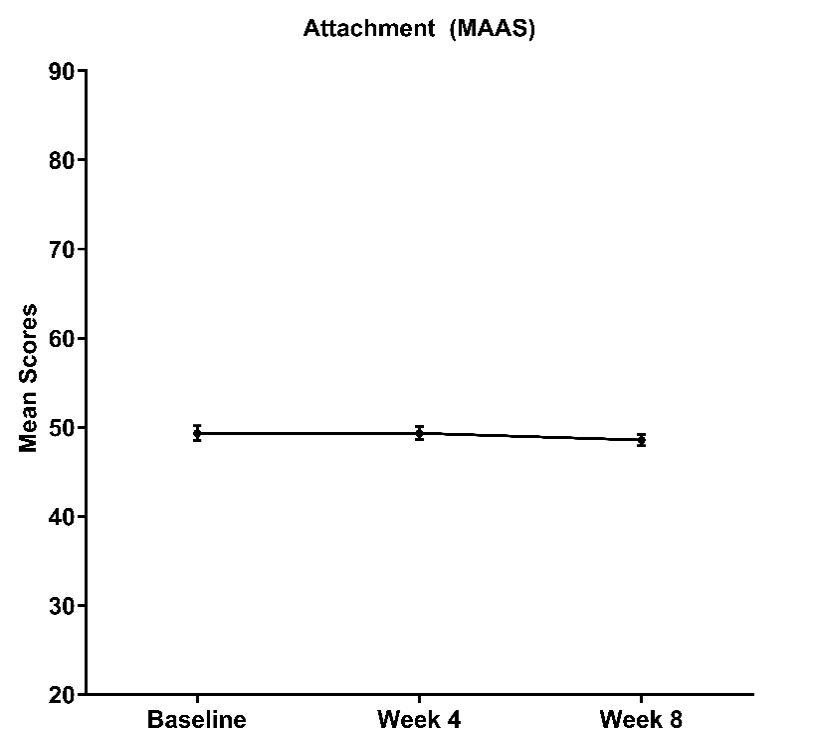


Figure 14 – Maternal antenatal attachment (MAAS Mean and SEM at baseline, week 4 and week 8). MAAS = Maternal Antenatal Attachment Scale; SEM = Standard Error of the Mean.

## Appendix VIII

Table 18 - Final Yoga Module tested in PRENAYOGA

| **Type of Practice** | **Practices** | **Instructions** |
| --- | --- | --- |
| Loosening/stretches | *Sukhasana*/side stretches | In easy pose, place your hand on the floor with your elbow slightly bent. Reach the opposite arm up  and overhead and lean over. |
|  | *Parivrtta sukhasana*/seated twist | From easy pose, twist upper body to one side while looking over the shoulder behind you. |
|  | Neck warm-up | Following the breath, twist the neck left-right, up-down, sideways and roll your neck in both directions. |
|  | Shoulder warm-up | Rotate your shoulders backwards and forwards while keeping the elbows bent and hands on shoulders. |
|  | Elbow stretches | Bend and release the arms, starting with the arms at shoulder height and finishing the movement with hands on shoulders and elbows pointing forward. |
|  | Wrist warm-ups | From tabletop, rotate around the wrists. Close the hands in fists, open them outward, and inward, with fingers facing up and down. |
|  | Loosening of fingers | Sitting on the ankles or in easy seat, close your fists and release them, opening the hands with arms straight in front. |
|  | Pelvic circles | Standing up with feet hip-width apart, rotate the waist clockwise and anti-clockwise. |
|  | Knee rotations | With the feet together, bend forward to touch the knees and rotate them clockwise and anti-clockwise. |
|  | Ankle rotations | Sitting with the legs straight in front of you, rotate the ankles clockwise and anticlockwise. |
|  | *Marjariasana-bitilasana*/cat-cow pose/tiger breathing | From tabletop, inhale, inflating the belly and dropping the spine towards the mat, exhaling curve the spine towards the ceiling and tuck the tailbone. |
| Asanas | *Tadasana*/mountain pose | Stand with feet slightly apart while keeping back straight. |
|  | *Trikonasana*/triangle pose | Legs 3 feet apart, open arms, lower bottom hand to knee or chin level. |
|  | *Ardhakati chakrasana*/half lateral arc pose | From *tadasana*, lift one arm above the head and bend the spine to the opposite side. |
|  | *Sukhasana*/easy seat pose | Seat with legs crossed ad spine straight. Use a block under the hips for comfort. |
|  | *Parvatasana*/seated mountain pose | In easy seat, raise arms above the head in *namaskara* *mudra* and stretch spine upwards. |
|  | *Vajrasana*/diamond pose | Sit with the legs bent and chins against the mat. The seat bones should rest on the heel and back should be straight. |
|  | *Titiliasana*/butterfly pose | Sit with the soles of the feet together and move knees up and down while keeping the back straight. Use a block under the hips if needed. |
|  | *Siddhasana*/accomplished pose | From easy seat, place hands on the knees in chin mudra and keep the arms straight. |
|  | *Parsva savasana* /side corpse pose | Lying on the back, roll to one side, bend your knees as necessary and place bolsters/blocks between the knees for support. Use lower arm on the floor to support head. |
| Meditation/chanting/pranayama (breathwork) | *Bhramari* *pranayama*/bumble bee breath | Close your eyes and hum during the exhale in an MMM sound. |
|  | *Nadi shodhana pranayama*/ alternate nostril breath | Place one hand in *chin mudra* and one hand in *nasika mudra*. Inhale from one nostril while closing the other; exhale from the opposite nostril. |
|  | *Ujjayi pranayama*/ victorious breath | Constrict the back of the throat to the point that the breath makes a rushing noise and keep inhalations and exhalations equal in duration. |
|  | Nadanusandhana pranayama | Chant separately A, U and M, and contemplate the vibrations in produces in the body. |
|  | Mindfulness meditation on breath and sensations | Please allow your breathing to settle down for you. As you inhale through your nose, feel your abdomen expanding out, and as you exhale, draw your navel towards your spine.  You have nowhere to be, nothing else to do.  With every inhalation, consciously slow down your breath, and bring awareness to the space around your nostrils. With every exhalation, draw your belly in, and let go of all your thoughts or any external distractions that are occupying your mind at this point.  Feel the cool air coming into your nostrils as you inhale, and the warm air leaving your nostrils as you exhale.  Continue with your inhalation and exhalation, slowly feeling a sense of steadiness and stability.  As you exhale, slowly open your eyes. |
|  | *Japa* meditation/mantra meditation | Meditative repetition of a mantra, silently, as you inhale and exhale. Use mantras such as “In me, I trust”, “Disconnect to reconnect“, “Everything I need is within me” or “I can and I will”.  Example - As you inhale, repeat in your mind "in me", as you exhale, repeat in your mind "I trust". |
|  | *Pranav* meditation/Om meditation breathing | Focus on the sound of Om while breathing normally in and out through the nostrils.  Sit in a cross-legged comfortable position, close your eyes & put your hands in a mudra (chin mudra is a good start).  At first, focus on your normal breathing by observing the breath entering & leaving at the nostrils.  Now when you come in tune with your normal breathing, draw an imaginary picture of Om Symbol in between the eyebrow centre, at the place of the third eye.  As you inhale, imagine you’re directing air to this Om at your eyebrows centre. As you exhale, imagine the same illumination of Om diminishing little by little & thus feel the calmness in mind now.  Mentally you can chant the sound of Om on every inhale & exhale. |
|  | Guided visualisation on baby-related themes | Imagine yourself holding the baby in your arms.  Imagine the weight of their little body, their warmth against your skin.  Imagine the smell of the top of their head and the feel of their skin against your lips as you kiss them.  Imagine their eyes looking up at you, into your eyes.  Imagine the sound of their voice and a wide smile on their face as they look at you.  With your eyes still closed, smile back at your baby. |
|  | Guided visualisation/body scan | Take a slow and deep breath in, bringing your attention to your expanding belly as you draw that air in. And release your breath on the exhale, breathing all the way out until you’re at the very end of your breath. Feel your belly get closer to your baby as it contracts in the exhale. On each inbreath, breathe in calm energy, feeling it soften your body and soften your mind. And on each outbreath, breathe out any tension, stress or discomfort. Go ahead and place your hands on your belly. As you do, recognise the warmth of the palm of your hands. Imagine the warmth of your hands travelling into your belly. Watch how tenderly it embraces your little one. And exhale, feeling calm and relaxed.  Perhaps you’re feeling heavy, nauseous, worried, tired or in pain. Take a moment to bring to mind all the discomforts you’ve been feeling during your pregnancy. Recognise them and visualise yourself letting putting them on a fluffy cloud above you and see the cloud float away from you into the sky. Feeling yourself expand and contract as you breathe, Perhaps even feeling your little one move around inside of you. And it’s complicated, detailed work. So over the next minute, breathe in some grace for your body, appreciation and love for what it’s doing for you and your baby; bringing this beautiful being into the world will require much of you. But you have everything you need to do it. If you’re feeling any fear or anxiety or worry about delivering your baby, think about another mum you know. Imagine her putting her hand on your shoulder, reminding you it’s all going to be okay. And as you breathe out, watch as she sits next to you. Now watch as another mom, perhaps your mom, or your sister, aunt or grandma, puts her hand on your shoulder and whispers to you, you are so strong. And as you breathe out, watch as she sits next to you.  You are not alone. You have all these women in this room sharing this moment with you.  You are all doing an amazing job. |
|  | Yoga *nidra*/guided deep relaxation | Lie down in a comfortable position and cover yourself with a blanket. Close your eyes and take three deep breaths. We'll do it together.  You can slow down your breath and turn your attention inward.  We are going to slowly scan your body, body part by body part.  Right-hand thumb … 1st finger … 2nd finger … 3rd finger … 4th finger … palm of the hand … back of the hand …wrist … forearm … elbow … upper arm … right shoulder … armpit … chest … waist… hip … groin … buttock … thigh … knee … calf … ankle … heel … sole of the foot… top of the foot … right big toe … 2nd toe … 3rd toe … 4th toe … 5thtoe. Left-hand thumb … 1st finger … 2nd finger … 3rd finger … 4th finger …palm of the hand … back of the hand … wrist … forearm … elbow … upper arm …left shoulder … armpit … chest … waist … hip … groin … buttock … thigh … knee… calf … ankle … heel … sole of the foot … top of the foot … left big toe …2nd toe … 3rd toe … 4th toe … 5th toe. Move your awareness to the top of the head … forehead … right temple … left temple … right ear … left ear … right cheek … left cheek … right eyebrow … left eyebrow … eyebrow centre… right eye … left eye … right nostril … left nostril … whole nose … upper lip … lower lip … chin … jaw … throat … right collarbone …left collarbone … right chest … left chest … heart centre… upper abdomen … navel … lower abdomen … right groin … left groin … the pelvic floor … tailbone … sacrum … right buttock …left buttock … the entire spine, from the tailbone to the base of the skull …right shoulder blade … left shoulder blade … back of the neck … back of the head … crown of the head. Now feel the whole right arm … the whole left arm …both arms together … the whole right leg … the whole left leg … both legs together … the entire torso … the face … the head … the body … the whole body… your entire body. |
| Yogic counselling | 4 Yoga streams to deal with adversity (*jnana*, *bhakti*, *raja* and *karma*) | The four paths of yoga (*Bhakti, Karma, Jnana*, and *Raja*) are all aimed at helping individuals achieve spiritual enlightenment and a more fulfilling life experience. Each path offers a unique approach and method, and it is important for individuals to find the path that resonates with them the most.  *Bhakti* Yoga – The Path of Devotion: *Bhakti* Yoga is the path of devotion. It is a practice that is centred on cultivating a deep and unwavering devotion to a deity or a personal god. *Bhakti* Yoga has many benefits, including increased devotion and faith, heightened spiritual connection, and a more fulfilling life experience.  *Karma* Yoga – The Path of Action: *Karma* Yoga is the path of action. It is a practice that emphasises the importance of selfless action and encourages individuals to perform good deeds without expecting anything in return. *Karma* Yoga has many benefits, including increased selflessness, reduced stress and anxiety, and a more fulfilling life experience. This can be as simple as volunteering at a local food bank or helping an elderly neighbour with their groceries.  *Jnana* Yoga – The Path of Wisdom: *Jnana* Yoga is the path of wisdom. It is a practice that emphasises the importance of self-awareness and self-discovery. *Jnana* Yoga has many benefits, including increased self-awareness, a deeper understanding of one’s place in the world, and a more fulfilling life experience. This can be achieved through practices such as meditation, journaling, and self-study.  *Raja* Yoga – The Royal Path: *Raja* Yoga is a practice that emphasises the importance of meditation and control of the mind. *Raja* Yoga has many benefits, including increased mental clarity, reduced stress and anxiety, and a more fulfilling life experience. To practice Raja Yoga, one must first focus on mastering the control of their mind through practices such as meditation and pranayama. |
|  | *Panchakosha* model (5 layers of existence) | Yoga philosophy teaches that the individual spirit or *atman* operates through five bodies or layers called the *pancha* *kosha*. It's like an onion with 5 layers, each one deeper than the last.  You are a multi-layered being, from your physical body to your subtle bliss body. The physical, tangible body is only your external self, like your outer peel. Stripping away this layer exposes a slightly more subtle layer, the *pranic* or energetic body. One more layer inward is the mental body, where all the thoughts are, then the intellectual body, where your knowledge about the world is stored, and finally, the bliss body, where ultimate happiness is.  In a yoga practice, we begin to bring greater integration to the physical body with yoga *asanas* and to the energetic body with yogic breathing or pranayama.  As the flow of vital energy is freed up in the physical body and breath body, this, in turn, creates greater vitality and integration in our mental-emotional body. This is why many people find that practicing yoga considerably improves their mood and well-being. |
|  | Connection exercises for mother-foetus (in the womb) | Next time you are at home and want to feel close to your baby, talk and sing to your them, knowing they can hear you. From 20 weeks they can see light and hear sounds, they recognise your voice and music you might play in the house.  You can also gently touch and rub your belly or massage it with oil in circular motions. Respond to your baby's kicks by pressing back where they kicked, like you’re playing a game with them or communicating with them. Any time you touch your belly, sing to or move your body with the intention to connect with your baby, your baby can feel it. |
|  | Affirmations | Affirmations during class, a selection from: “I trust my body”, “I will have a healthy and happy baby”, "I accept things as they are, I open my arms to what life brings”, “my baby is going to be a great human being”, “my baby is going to be strong, content, collected”, “I am prepared for this”, “I have done my best”, “I trust my body; my body knows what to do”, “I surrender to what’s to come, I am prepared to handle it”. |
|  | Cultivate gratitude for their bodies, their babies, the time they've taken to do yoga (in *namaskara mudra*) | Cultivate gratitude for their bodies that are working overtime to grow a healthy and happy baby, their babies that are so lucky to have them as mothers, and the time they've taken to do yoga to nourish their bodies and their minds. Feel gratitude for all the other mothers in the room, going through the same journey and them, showing them that they are not alone. You can do this in *namaskara* *mudra* for example. |
|  | Concepts of maitri, karuna, mudita, and upeksha | By embracing the 4 Immeasurables as discussed in Pantanjali’s Yoga Sutras, we have a wonderful support system that encourages positive attitudes and behaviors to create a fulfilling relationship:  *Maitri* (loving kindness) is a gentle, loyal acceptance with a deep sense of appreciation and regard.  *Karuna* (compassion) is the intention and capacity to relieve and transform suffering to lighten sorrow.  *Mudita* (joy) is tue love brings joy, and *mudita* is the joy we take in simple pleasures. When we love, joy seems to surround and pervade us”.  *Upeksha* (equanimity) is the ability to feel  a connection fully, without clinging or possessiveness. |
|  | Yamas and niyamas | The first two limbs of yoga, *yamas: satya* (truthfulness), *ahimsa* (non-violence), *asteya* (non-stealing), *bramacharya* (self-restraint) and *aparigraha* (non-hoarding) and *niyamas*: *saucha* (purity), *santosha* (contentment), *tapas* (penance), *swadhyaya* (self-study) and *ishwara pranidhana* (surrender to a higher force).  The practices of *yamas* cuts down the incessant flow of desires and resulting mental turbulence. The *niyamas* give directions to an individual to how to channel their energy and efforts. Particularly for the improvement of mental health one should practice:  - *Asteya*: one must be content with their circumstances,  - *Santosha*: one must accept with joy what life brings  - *Swadhyaya*: one should reflect on their thought patterns, emotions and stage of learning. |
|  | Yoga for labour | Contractions:  Sit in *badha konasana* and exhale during contractions; between contractions, and practice relaxation techniques.  Move/walk around the delivery room between contractions, and can also sit in *malasana*.  Breathing:  Breathe using *ujjayi pranayama* to lengthen and deepen breaths. Affirmations: "I am dilating", "the birth is progressing", "my baby is ready to arrive". |

Table 19 - Adaptations resulting from interviews with stakeholders (yoga teachers)

| **Type of Practice** | **Practices** | **Justification** | **Teacher** |
| --- | --- | --- | --- |
| Asana | *Utkata Konasana, Virabhadrasana* I and II | Incorporate more standing postures, especially in afternoon classes, to boost energy and strengthen legs. Avoid overly dynamic sequences but focus on gentle strengthening. | Teacher #1 |
| Chanting | Internal chanting or recorded chanting | Offer culturally sensitive alternatives such as internal chanting ("in the mind’s eye") or silent mantra repetition. Play recorded chanting music for 2 minutes if appropriate. | Teacher #1 |
| Adaptability Protocols | Restorative poses and kindness-focused breathing | Provide restorative options and soft, kindness-focused breathing techniques for participants needing emotional support. | Teacher #1 |
| Asana | *Malasana* | Introduce props such as bricks for support and use walls for transitions, e.g., moving from *malasana* to standing | Teacher #2 |
| Dynamic Movements | Flowing, movement-based postures | Encourage flowing movements instead of static postures to adapt to the changing needs of pregnant participants. | Teacher #2 |
| Participant-Centric Approach | Encouraging self-exploration | Guide participants to move intuitively and find personalised comfort and alignment in poses. | Teacher #2 |
